# A polysaccharide utilization locus from the gut bacterium *Dysgonomonas mossii* encodes functionally distinct carbohydrate esterases

**DOI:** 10.1016/j.jbc.2021.100500

**Published:** 2021-03-02

**Authors:** Cathleen Kmezik, Scott Mazurkewich, Tomke Meents, Lauren Sara McKee, Alexander Idström, Marina Armeni, Otto Savolainen, Gisela Brändén, Johan Larsbrink

**Affiliations:** 1 Division of Industrial Biotechnology, Department of Biology and Biological Engineering, Chalmers University of Technology, Gothenburg, Sweden; 2 Division of Glycoscience, Department of Chemistry, Royal Institute of Technology (KTH), AlbaNova University Centre, Stockholm, Sweden; 3 Wallenberg Wood Science Center, Stockholm, Sweden; 4 Department of Chemistry and Chemical Engineering, Chalmers University of Technology, Gothenburg, Sweden; 5 Chalmers Mass Spectrometry Infrastructure, Department of Biology and Biological Engineering, Chalmers University of Technology, Gothenburg, Sweden; 6 Department of Clinical Nutrition, Institute of Public Health and Clinical Nutrition, University of Eastern Finland, Kuopio, Finland; 7 Department of Chemistry and Molecular Biology, University of Gothenburg, Gothenburg, Sweden

**Keywords:** serine esterase, enzyme kinetics, crystal structure, enzyme structure, carbohydrate-binding protein, polysaccharide utilization locus, carbohydrate-active enzyme, multidomain enzymes, acetyl xylan esterase, feruloyl esterase, 4-MU-Ac, 4-methylumbelliferyl acetate, CAZy, carbohydrate-active enzyme database, CAZyme, carbohydrate-active enzyme, CBM, carbohydrate-binding module, CE, carbohydrate esterase, CE1, carbohydrate esterase family 1, CE6, carbohydrate esterase family 6, COSY, correlation spectroscopy, DMSO, dimethyl sulfoxide, DNSA, 3,5-dinitrosalicylic acid, FA, ferulic acid, FAE, feruloyl esterase, GAX, glucuronoarabinoxylan, GH, glycoside hydrolase, HMBC, heteronuclear multiple bond correlation, HPAEC-PAD, high-performance anion-exchange chromatography with pulsed amperometric detection, HSQC, heteronuclear single quantum coherence spectroscopy, IMAC, immobilized metal ion affinity chromatography, IPTG, isopropyl-β-d-1-thiogalactopyranoside, LB, lysogeny broth, LC-MS/MS, liquid chromatography with tandem mass spectrometry, MCA, methyl caffeate, MFA, methyl ferulate, M*p*CA, methyl *p*-coumarate, MSA, methyl sinapate, NMR, nuclear magnetic resonance, PDB, protein data bank, *p*NP-Ac, *p*-nitrophenyl acetate, PUL, polysaccharide utilization locus, SCFAs, short chain fatty acids, SDS-PAGE, sodium dodecyl sulfate–polyacrylamide gel electrophoresis, Sus, starch utilization system, TetAcXyl, 1,2,3,4-tetra-*O*-acetyl-d-xylopyranose, UHPLC-HRMS, ultraperformance liquid chromatography–high-resolution mass spectrometry, X_1-6_, xylose, xylobiose, xylotriose, xylotetraose, xylopentaose, and xylohexaose, respectively, XO, xylooligosaccharide, XylUL, xylan utilization locus

## Abstract

The gut microbiota plays a central role in human health by enzymatically degrading dietary fiber and concomitantly excreting short chain fatty acids that are associated with manifold health benefits. The polysaccharide xylan is abundant in dietary fiber but noncarbohydrate decorations hinder efficient cleavage by glycoside hydrolases (GHs) and need to be addressed by carbohydrate esterases (CEs). Enzymes from carbohydrate esterase families 1 and 6 (CE1 and 6) perform key roles in xylan degradation by removing feruloyl and acetate decorations, yet little is known about these enzyme families especially with regard to their diversity in activity. Bacteroidetes bacteria are dominant members of the microbiota and often encode their carbohydrate-active enzymes in multigene polysaccharide utilization loci (PULs). Here we present the characterization of three CEs found in a PUL encoded by the gut Bacteroidete *Dysgonomonas mossii*. We demonstrate that the CEs are functionally distinct, with one highly efficient CE6 acetyl esterase and two CE1 enzymes with feruloyl esterase activities. One multidomain CE1 enzyme contains two CE1 domains: an N-terminal domain feruloyl esterase, and a C-terminal domain with minimal activity on model substrates. We present the structure of the C-terminal CE1 domain with the carbohydrate-binding module that bridges the two CE1 domains, as well as a complex of the same protein fragment with methyl ferulate. The investment of *D. mossii* in producing multiple CEs suggests that improved accessibility of xylan for GHs and cleavage of covalent polysaccharide-polysaccharide and lignin-polysaccharide bonds are important enzyme activities in the gut environment.

The gastrointestinal tract of mammals is a complex environment characterized by very high densities of microorganisms. The majority of the human gut microbiota is located in the colon (1), where bacterial communities degrade undigested food particles (dietary fiber), which the human host cannot directly process (2). In return, many species found in these microbial communities secrete short chain fatty acids (SCFAs), which can be absorbed and metabolized by host cells (3). The major SCFAs released are acetate, propionate, and butyrate (4). Multiple health benefits have been ascribed to SCFAs, and the link between dietary fiber and SCFA production has led to recommendations of a daily intake of 12.5 g of dietary fiber per 1000 calories (5, 6). A deficit or imbalance in SCFAs has been associated with various inflammatory and autoimmune diseases such as Crohn’s disease and colon cancer (7). The microbiota of the human colon in Western population groups is typically dominated by bacteria belonging to the phyla Bacteroidetes and Firmicutes (8).

While Firmicutes have only recently received increased attention regarding their carbohydrate-active enzyme (CAZyme) repertoires (9), members of the Bacteroidetes phylum have long been recognized for their ability to metabolize complex polysaccharides using setups of distinct gene clusters, so-called polysaccharide utilization loci (PULs) (10, 11). The first PUL described was the archetypical starch utilization system (Sus) (12, 13), which now serves as a general template for similar systems. PULs encode sugar capture- and transport proteins (SusD-like and SusC-like, respectively), sensory/regulatory proteins enabling a rapid response to carbohydrates in the cell’s surroundings, as well as various combinations of CAZymes to enable targeted degradation of a specific polysaccharide. By mining for conserved SusC/SusD-like sequences in other genomes, PULs can be predicted and are collected in the PULDB database (www.cazy.org/PULDB; (14)) within the larger carbohydrate active enzymes database (CAZy; www.cazy.org; (15)). A range of PULs have been characterized to date and shown to enable metabolism of a large variety of glycans from both plant and microbial sources (16, 17, 18, 19, 20).

In the Bacteroidetes phylum, the genus *Dysgonomonas* forms a phylogenetic cluster within the *Bacteroides-Prevotella-Porphyromonas* group, and the genus maintains genome sequence similarities between 79 and 88% to the other group members (21). *Dysgonomonas mossii* is a species that was first isolated from human abdominal drainage, and it has been demonstrated to hydrolyze starch and produce SCFAs from polysaccharide building blocks such as l-arabinose, cellobiose, l-rhamnose, and d-xylose (21). The capabilities of *D. mossii* to degrade complex carbohydrates have not been studied in detail, but according to the PULDB, it encodes 29 putative PULs, none as of yet supported with experimental data. The predicted activities of the putative CAZymes found in the largest predicted PUL of *D. mossii*, PUL 17, suggest that this bacterium is able to target complex xylan polysaccharides that are abundant in dietary fiber.

Xylan is a major plant cell wall component and is the second most abundant polysaccharide in hardwoods and grass species after cellulose (22). The backbone of xylan consists of *β*-1,4-linked xylose moieties that can be substituted with various carbohydrate and noncarbohydrate decorations depending on the plant species and tissue (23). Glucuronoarabinoxylan (GAX) is the most structurally complex form of xylan, requiring multiple enzymatic activities for complete hydrolysis into monosaccharides. Carbohydrate decorations on the main chains of GAX include *α*-1,2-linked d-glucuronic acid, *α*-1,2 and/or *α*-1,3 linked l-arabinofuranosyl groups, and *α*-1,2-, *α*-1,3- and/or *β*-1,3-linked xylosyl substitutions (24). Noncarbohydrate decorations include acetylation at the *O*-2 and/or *O*-3 positions of the xylan backbone (25, 26) and feruloyl decorations of arabinofuranosyl sugars (5-*O*-*trans*-feruloyl-l-arabinofuranosyl) (27, 28). Feruloyl groups can additionally be either linked to lignin or neighboring feruloylated GAX chains by forming diferulate cross-links that increase recalcitrance to enzymatic degradation (27, 28, 29). Specifically, noncarbohydrate decorations on the xylan backbone have been shown to inhibit the activity of *endo*-xylanases, thus impeding efficient biomass degradation (30, 31). The enzyme repertoires used by mammalian gut symbionts could therefore also serve as a source for the discovery of enzymes relevant to biorefineries.

Carbohydrate esterases (CEs) are enzymes able to remove ester-linked decorations from carbohydrates. Enzymes from carbohydrate esterase family 1 (CE1) and CE6 are common in PULs and have been shown, or predicted, to target xylans (14). CE6 enzymes have exclusively been shown to be acetyl xylan esterases, though only a few enzymes from the family have been characterized to date (15). Several specificities have conversely been discovered in CE1, such as acetyl xylan esterase, feruloyl esterase, and cinnamoyl esterase. Specificity toward certain feruloylations and whether the heterogeneity of lignin influences feruloyl esterase activity is thus far not well understood. In the densely colonized gut, there is fierce competition for nutrients, and it is likely that possession of efficient CEs that enable greater access to cell wall polysaccharides can give microorganisms a competitive advantage. In a recent study, we demonstrated that in two unique PULs from the gut bacterium *Bacteroides ovatus* and the aerobic soil bacterium *Flavobacterium johnsoniae*, CE1 and CE6 enzymes are found in “multicatalytic” enzymes, *i.e.*, enzymes comprised of multiple catalytic domains on the same polypeptide (32). The combination of complementary enzymatic activities into single proteins with strongly enhanced activity has previously been shown mainly for enzyme activities targeting the main polysaccharide chain (20, 33), but research on multicatalytic enzymes is often impeded by their large size and resulting difficulties in protein production (34). The benefit from combining “accessory” CE activities into a single enzyme is not as obvious as for glycoside hydrolases (GHs), where *e.g.*, an *endo*-acting enzyme can provide new chain ends for *exo*-acting enzymes that in turn reveal new sites of attack for the *endo*-acting enzymes and so forth. Combined CE action may instead “only” trim carbohydrate decorations. Why a simultaneous attack by fused enzymes is desirable is not clear. However, we recently showed that CE action may also be enhanced by a multicatalytic protein architecture when aiding *endo*-acting GH enzymes (32).

In the present work, we have investigated the role of the CEs encoded in PUL 17 of the gut bacterium *D. mossii*. The PUL, assumed to target xylan, encodes one CE6 and two CE1 enzymes, one of which was shown to be a multicatalytic CE1-CE1 fusion. Studies of such CE1-CE1 fusions have to our knowledge not previously been reported. The enzymes were biochemically characterized on model substrates by kinetic studies and product analyses. The multicatalytic CE1 enzyme was studied in both its full-length form and as truncated single catalytic domain variants. Both CE1 enzymes contain carbohydrate-binding modules (CBMs) from family 48 (CBM48), though binding studies of these revealed no affinity to the range of glycans tested. Boosting studies where the enzymes were used in conjunction with a xylanase were performed on milled corn cob biomass as a native substrate, with the results supporting the hypothesis that each PUL-encoded CE serves a distinct biological function in the xylan metabolism of *D. mossii*. We further present the structure of the C-terminal CE1 and internal CBM48 domains of the multicatalytic *Dm*CE1B enzyme. The structure was solved both in the apo form and with a feruloyl esterase substrate ligand, and these provide a basis of understanding the elusive substrate specificity of this catalytic domain.

## Results

### The predicted PUL 17 of *D. mossii* encodes multiple carbohydrate esterases

**Figure 1:**
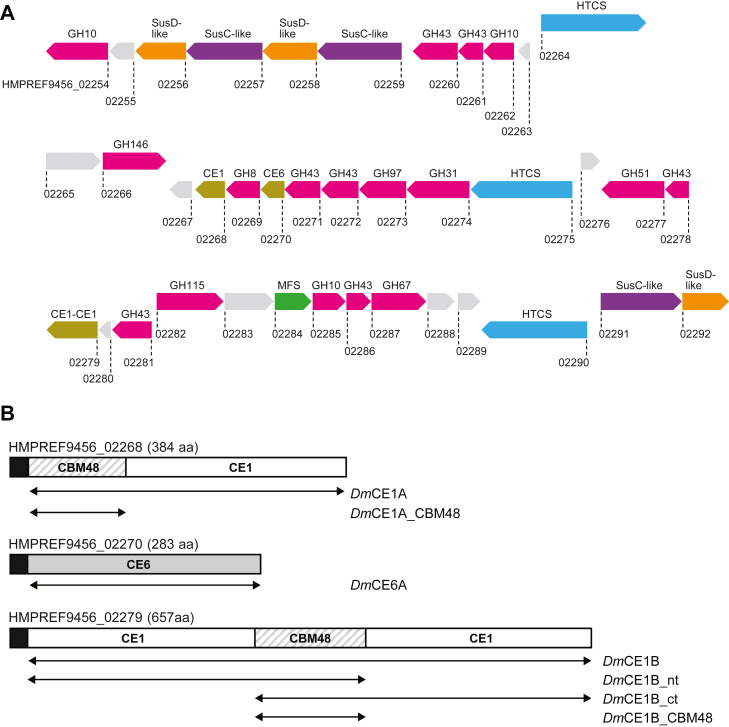
**PUL of *D. mossii* encoding the studied carbohydrate esterases and their enzyme architectures.***A*, the predicted PUL of *D. mossii* spans 39 genes (locus tags HMPREF9456_02254–02292), the products of which have not previously been characterized. Locus tags are shown below each protein. Functional annotations are shown above each gene symbol and are color coded: GHs (*pink*), proteins of unknown function (*light grey*), SusD-like binding proteins (*orange*), SusC-like transport proteins (*purple*), regulatory hybrid two-component systems (HTCSs; *blue*), CEs (*brown*), and major facilitator family transporters (MFSs; *green*). *B*, protein architecture of *Dm*CE1A (HMPREF9456_02268), *Dm*CE6A (HMPREF9456_02270), and *Dm*CE1B (HMPREF9456_02279) and their truncated versions used here. Predicted N-terminal signal peptides are marked in *black*.

Of the 29 predicted PULs found in the *D. mossii* genome, 19 are composed of six or fewer proteins, and several only encode a single SusC/D-like pair (PULDB; (14)) and may not be functional PULs targeting polysaccharides. The largest and most complex of the *D. mossii* PULs is labeled PUL 17 (locus tags HMPREF9456_02254–HMPREF9456_02292; Fig. 1*A*), and none of its 39 encoded proteins have previously been biochemically characterized. The PUL encodes glycoside hydrolases (GHs) from families known to target xylan, including putative xylanases (GH8, GH10), a *β*-xylosidase (GH43), *α*-l-arabinofuranosidases (GH43, GH51, GH146), and *α*-glucuronidases (GH67, GH115), which collectively indicate that PUL 17 likely targets complex GAX. This is further supported by the fact that the PUL also contains a set of CE1 and CE6 enzymes (*Dm*CE1A, *Dm*CE1B, and *Dm*CE6A), which may enable removal of acetyl and feruloyl moieties, which are common in GAX. The listed enzyme families are also present in the characterized xylan-targeting PUL *Bo*XylL, which enables *B. ovatus* to metabolize complex GAX (24). The overall architecture of the *D. mossii* PUL17 however appears unique, and no similar PULs can be found currently in the PULDB (14). Our recent biochemical characterization of the *Bo*XylL-encoded multicatalytic *Bo*CE6-CE1 enzyme showed how it could greatly boost the GAX degradation ability of a commercial xylanase through its combined acetyl- and feruloyl esterase activities (32). Interestingly, *Dm*CE1B in the *D. mossii* PUL 17 comprises two individual CE1 catalytic domains separated by a small CBM48 domain (Fig. 1*B*). While numerous putative CE6-CE1 fusions can be found in the PULDB, only two have been characterized (32). As mentioned, no CE1-CE1 fusion enzymes have previously been characterized, though such enzymes can be found in various *Dysgonomonas* species and other genera from both gastrointestinal and environmental habitats including *Chryseobacterium*, *Dyadobacter*, and *Chitinophaga* (14).

### Analysis of the primary structures of the *D. mossii* esterases

Both *Dm*CE1A and *Dm*CE1B are multidomain enzymes, where *Dm*CE1B as mentioned comprises two CE1 domains bridged by a CBM48 domain, while *Dm*CE1A has a single CE1 domain and an N-terminal CBM48 domain (Fig. 1*B*). Comparative amino acid sequence analysis against sequences from the Protein Data Bank (PDB; (35)) revealed that the CE1 domains of these enzymes shared the highest sequence similarity with feruloyl esterases with sequence identities of 43 to 53% to studied enzymes (Table S1). Compared with each other, the *D. mossii* CE1 domains displayed moderate similarities: *Dm*CE1A shares 36% and 49% sequence identity with the N- and C-terminal CE1 domains of *Dm*CE1B, respectively. The two CE1 domains in *Dm*CE1B share 35% sequence identity, illustrating that they are not closely related and likely *Dm*CE1B has not arisen from a recent gene duplication event of one of the two domains. A multiple sequence alignment of the CE1 domains investigated here with sequences of characterized feruloyl esterases revealed that each *D. mossii* CE1 domain contained the expected conserved Ser-His-Asp/Glu catalytic triad (Fig. S1). The CE6 enzyme of PUL 17 is composed of a single catalytic domain (Fig. 1*B*). *Dm*CE6A had relatively high sequence identity to the previously characterized acetyl xylan esterase *Fj*CE6 from *F. johnsoniae* (seq. id. 63%) (32), and also in this enzyme the esterase catalytic triad appeared conserved based on a multiple sequence alignment (Fig. S2).

Signal peptide predictions using SignalP (36) showed that *Dm*CE1A, *Dm*CE1B, and *Dm*CE6A all possess putative N-terminal Sec/SPI signal peptides and are thus predicted to be secreted. Whether the proteins reside in the periplasm or are further exported outside of the cell is not known. Since only a small number of CE1 and CE6 members have been thoroughly characterized to date, and knowledge on multicatalytic CEs is still very limited, we pursued biochemical characterization of the various CE domains found in PUL 17 to confirm their predicted activities and shed light on their roles in the biology of *D. mossii*.

### Biochemical characterization on model substrates supports sequence-based predictions

The different CEs of the *D. mossii* PUL 17 were cloned and heterologously produced in *E. coli* resulting in the enzymes *Dm*CE1A (43.5 kDa), *Dm*CE1B (73.5 kDa), and *Dm*CE6A (31.7 kDa; Fig. S3). To assess the role of each catalytic domain of the multicatalytic *Dm*CE1B, the separate domains were also produced: *Dm*CE1B_nt (45.3 kDa), comprising the N-terminal CE1 domain and the CBM48 domain, and *Dm*CE1B_ct (43.3 kDa) comprising the CBM48 domain and the C-terminal CE1 domain (Fig. 1*B*). All enzymes were assayed on model substrates to test for acetyl esterase activity (4-methylumbelliferyl acetate, 4-MU-Ac; and *p*-nitrophenyl acetate, *p*NP-Ac) and feruloyl esterase activity (methyl ferulate, MFA; methyl sinapate, MSA; methyl caffeate, MCA; and methyl *p*-coumarate, M*p*CA). The kinetic parameters of all enzyme constructs were determined where possible and are shown in Table 1.

**Table 1:** Kinetic parameters of investigated carbohydrate esterases where activity could be detected

Enzyme	Substrate	*K*_M_ (mM)	*k*_*cat*_ (s^−1^)	*k*_*cat*_/*K*_M_ (s^−1^ mM^−1^)
*Dm*CE1A	*p*NP-Ac	4.4 ± 0.67	1.03 ± 0.07	0.23 ± 0.04
	4-MU-Ac	0.55 ± 0.06	38.6 ± 1.26	70 ± 8
	MFA	not saturable up to 0.4 mM		0.12 ± 0.007
	MSA	0.18 ± 0.04	0.13 ± 0.01	0.70 ± 0.2
*Dm*CE1B	*p*NP-Ac	3.5 ± 0.70	12.92 ± 1.1	3.7 ± 0.81
	4-MU-Ac	0.37 ± 0.06	870 ± 3	2300 ± 400
	MFA	not saturable up to 0.4 mM		5.0 ± 0.35
	MSA	0.36 ± 0.10	5.0 ± 0.83	14 ± 4.5
	M*p*CA	not saturable up to 0.4 mM		0.59 ± 0.02
*Dm*CE1B_nt	*p*NP-Ac	1.2 ± 0.24	6.0 ± 0.40	5.1 ± 1.1
	4-MU-Ac	0.30 ± 0.04	630 ± 22	2100 ± 300
	MFA	0.16 ± 0.03	1.8 ± 0.13	11 ± 2.0
	MSA	0.29 ± 0.07	2.7 ± 0.34	9.2 ± 2.4
	M*p*CA	0.31 ± 0.09	0.61 ± 0.10	2.0 ± 0.7
*Dm*CE1B_ct	*p*NP-Ac	3.8 ± 0.77	0.15 ± 0.0015	0.041 ± 0.009
	4-MU-Ac	0.42 ± 0.10	11 ± 0.71	26 ± 6.6
*Dm*CE6A	*p*NP-Ac	0.18 ± 0.01	59 ± 0.87	320 ± 20
	4-MU-Ac	0.029 ± 0.003	38 × 10^3^ ± 1200	1.3 × 10^6^ ± 2 × 10^5^

All carbohydrate esterases were assayed on *p*NP-Ac, 4-MU-Ac, MFA, MSA, MCA, and M*p*CA. Kinetic parameters are shown for reactions where activity was detected and possible to measure. The data represent triplicate measurements, fitted to the Michaelis–Menten equation using OriginPro software, and presented as average with standard errors of mean. The catalytic efficiency (*k*_cat_/*K*_M_) was determined using linear regression for reactions that could not be saturated without exceeding the detection limit.

*Dm*CE1A catalyzed the cleavage of both acetyl esterase model substrates but with a 300-fold higher catalytic efficiency on 4-MU-Ac *versus p*NP-Ac (*k*_*cat*_/*K*_*M*_ of 70 and 0.23 s^−1^ mM^−1^, respectively). *Dm*CE1A had only minimal feruloyl esterase activity on the model substrates MFA and MSA. Interestingly, the enzyme had a higher activity on the more substituted MSA than on MFA, while not having any detectable activity on MCA and M*p*CA. It should be noted that minimal activity on feruloyl esterase model substrates does not rule out possible feruloyl esterase activity on natural substrates, as the former may only poorly mimic the complexity of the actual substrates in the plant cell wall. *Dm*CE1A was fully active over a broad pH range, between pH 6.0 and 7.5 (Fig. S4), consistent with the pH in the distal gut.

The full-length *Dm*CE1B was, similarly to *Dm*CE1A, active on both tested acetyl esterase substrates, with the catalytic efficiency on 4-MU-Ac being 30-fold higher than that of *Dm*CE1A (2300 *versus* 70 s^−1^ mM^−1^, respectively). *Dm*CE1B was also active on three out of the four tested feruloyl esterase substrates with the highest catalytic efficiency on MSA, followed by MFA and M*p*CA. Feruloyl esterases can often also be active on acetyl esterase model substrates, though the inverse is not the case (32), and it is therefore likely that *Dm*CE1B is a feruloyl esterase. Comparing the kinetic parameters for both *Dm*CE1A and *Dm*CE1B on MSA to other characterized CE1 members reveals that their *K*_*M*_ values on MSA (0.18 and 0.36 mM, respectively) are similar to those of the *Aspergillus niger An*FaeA and the *F. johnsoniae Fj*CE1 feruloyl esterases (0.24 and 0.14 mM, respectively) (32, 37). The turnover numbers of both *Dm*CE1A and *Dm*CE1B on MSA (0.13 and 5.0 s^−1^, respectively) are small relative to what is reported for *An*FaeA (85 s^−1^) but similar to that of *Fj*CE1 (1.8 s^−1^).

The two catalytic domains of *Dm*CE1B were studied separately to investigate whether they had different activities, as suggested by their difference in amino acid sequence. *Dm*CE1B_nt was active on the same model substrates as the full-length *Dm*CE1B and, interestingly, the catalytic efficiencies were very similar in general (Table 1). Conversely, no feruloyl esterase activity could be detected for *Dm*CE1B_ct and only minimal activity on the two acetyl esterase substrates, which were two orders of magnitude lower than the ones obtained for *Dm*CE1B and *Dm*CE1B_nt. *Dm*CE1B_ct was additionally tested on various cutinase substrates (*p*NP-butyrate, *p*NP-octanoate, *p*NP-dodecanoate, and *p*NP-palmitate) though no activity was detected. The results indicate that the observed activity of *Dm*CE1B on the model substrates stems largely from its N-terminal domain. The relatively low activity of *Dm*CE1B_nt on the feruloyl esterase substrates is thus not caused by its truncation into a single domain enzyme, and the superior activity of *Dm*CE1B over *Dm*CE1A on these model substrates is likely not an effect of its multicatalytic architecture. However, the different activity profiles of the three CE1 domains of *Dm*CE1A and *Dm*CE1B imply that each domain might fulfill a different biological role in the degradation of natural substrates in the gut. Under the conditions investigated here, *Dm*CE1B_nt had the highest activity at pH 6.5, *Dm*CE1B_ct at pH 8, and the full-length *Dm*CE1B at pH 7 (Fig. S4).

As expected from the sequence analysis, *Dm*CE6A was not active on any of the feruloyl esterase model substrates but instead had considerable activity on both acetyl esterase model substrates and was most active at pH 8 under the here investigated conditions (Fig. S4). 4-MU-Ac was a significantly better substrate for the enzyme compared with *p*NP-Ac, with *k*_*cat*_/*K*_*M*_ values of 1.3 × 10^6^ and 320 s^−1^ mM^−1^, respectively. The large difference stems from both a lower *K*_*M*_ value and a 650-fold higher *k*_*cat*_ on 4-MU-Ac. Notably, the catalytic efficiency of *Dm*CE6A on 4-MU-Ac was 30,000-fold higher than that of the previously reported *Fj*CE6 and *Bo*CE6 enzymes, mainly thanks to a turnover number four orders of magnitude higher than for the other two enzymes (32). This was an interesting observation as the sequence identity between *Dm*CE6A and *Fj*CE6, as mentioned previously, is relatively high. The catalytic efficiency of *Dm*CE6A on 4-MU-Ac was also two orders of magnitude higher than those reported for the bacterial acetyl xylan esterases Esterase I and Esterase II from *Thermoanaerobacterium* sp. strain JW/SL-YS485 on 4-MU-Ac (38).

### Carbohydrate esterases aid in xylanase hydrolysis of corn cob biomass

CEs are expected to act in concert with other enzymes to facilitate polysaccharide deconstruction. In PULs, other encoded GH or polysaccharide lyase (PL) CAZymes are the obvious partners of CEs, which remove noncarbohydrate decorations, thus facilitating access and depolymerization of the target polysaccharides or oligosaccharides. To probe the ability of the PUL 17 CEs to aid GH action, we used them in conjunction with a commercially available xylanase in the degradation of a complex natural substrate. For this we chose Xyn11A, a GH11 *endo*-1,4-*β*-xylanase originating from the fungus *Neocallimastix patriciarum*, which has previously been used to study how CEs can boost xylanase depolymerization of complex xylan substrates (32, 34, 39). Ball-milled and freeze-dried corn cob biomass (5% w/v) was used as substrate, as it contains significant amounts of complex GAX (24). The corn cob GAX is highly decorated with carbohydrate side groups as well as acetyl- and feruloyl substitutions (40, 41), which makes it a challenging substrate for enzymatic degradation.

**Figure 2:**
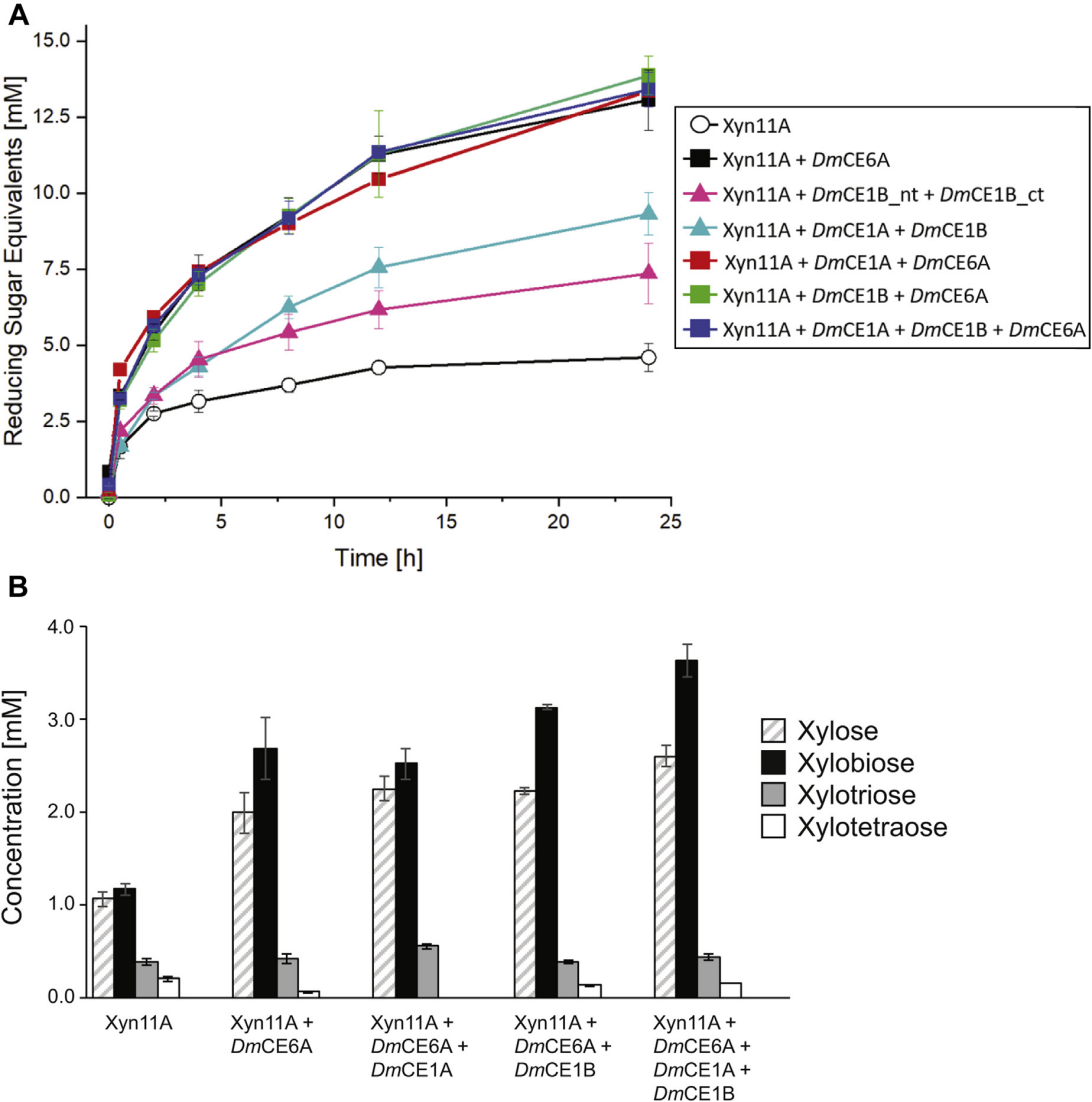
**Effect of esterase supplementation on the hydrolysis of corn cob biomass by a xylanase.***A*, reducing sugars were measured during the incubation of 5% w/v corn cob biomass (ball-milled and freeze-dried) at 37 °C with xylanase (Xyn11A) and carbohydrate esterase supplementation. *B*, xylooligosaccharide concentrations measured using HPAEC-PAD after 24 h reactions with Xyn11A and different carbohydrate esterases. Xylopentaose and xylohexaose were below quantifiable levels.

The hydrolysis reactions of corn cob xylan were followed over time using reducing sugar measurements (3,5-dinitrosalicylic acid, DNSA assay). Reactions containing only Xyn11A were used as a control reaction (Fig. 2*A*; reactions without added enzyme yielded no release of reducing sugars). Supplementation of Xyn11A with either *Dm*CE1A, *Dm*CE1B, *Dm*CE1B_nt or *Dm*CE1B_ct did not alter the amount of sugar released compared with the control (data not shown). Simultaneous supplementation of Xyn11A with both *Dm*CE1A and *Dm*CE1B together yielded a twofold increase in reducing sugar equivalents released after 24 h compared with the control reaction. Likewise, simultaneous supplementation with *Dm*CE1B_nt and *Dm*CE1B_ct led to a 1.7-fold increase of reducing sugar after 24 h, despite the lack of any observable effect from addition of the intact *Dm*CE1B.

Addition of *Dm*CE6A to the Xyn11A hydrolysis reaction resulted in a rapid increase of reducing sugars, with a twofold increase after 0.5 h and threefold after 24 h compared with the control reaction. The improved ability of Xyn11A to hydrolyze the corn cob biomass most likely stems from deacetylation of the xylan backbone by *Dm*CE6A, as has been reported previously for the *Orpinomyces* acetyl esterase AxeA combined with xylanase A from the same organism (42). In all Xyn11A reactions where *Dm*CE6A was present, a similar effect was seen (*i.e.*, Xyn11A + *Dm*CE6A, Xyn11A + *Dm*CE1B + *Dm*CE6A, Xyn11A + *Dm*CE1A + *Dm*CE6A, and Xyn11A + *Dm*CE1A + *Dm*CE1B + *Dm*CE6A; Fig. 2*A*). As indicated by the assays on model substrates, *Dm*CE6A is a strikingly efficient esterase and appears able to completely overshadow the activities of the CE1 enzymes under the experimental conditions, which utilized equimolar concentrations of all CE enzymes.

To further study the influence of the *D. mossii* CE1 enzymes on the xylanase hydrolysis of corn cob biomass, high-performance anion exchange chromatography with pulsed amperometric detection (HPAEC-PAD) was used to quantify released xylooligosaccharides (XOs): xylose (X_1_), xylobiose (X_2_), xylotriose (X_3_), xylotetraose (X_4_), xylopentaose (X_5_), and xylohexaose (X_6_) (Fig. 2*B*). When Xyn11A was not supplemented with CEs (control reaction), X_1_ and X_2_ were the most abundant XOs with concentrations of 1.1 mM and 1.2 mM, respectively, followed by X_3_ (0.4 mM) and X_4_ (0.2 mM). X_5_ and X_6_ were not detected. As expected from the DNSA assay results, supplementation of Xyn11A with *Dm*CE6A yielded a large difference in the XO profile with doubled X_1_ and X_2_ concentrations, unchanged X_3_ concentrations and X_4_ concentrations that were halved compared with the control reaction. Supplementation of Xyn11A and *Dm*CE6A with the CE1 enzymes resulted in subtle differences in the XO profiles. When adding *Dm*CE1A to the Xyn11A + *Dm*CE6A reaction, X_3_ concentrations increased, while X_4_ concentrations were reduced below the detection limit. Addition of *Dm*CE1B increased X_1_ and X_2_ concentrations slightly. Combination of *Dm*CE1A, *Dm*CE1B, *Dm*CE6A, and Xyn11A yielded the highest X_1_ and X_2_ concentrations, reaching 2.6 mM and 3.6 mM, respectively. These results indicate that a combination of all of the PUL 17 CEs may best aid the xylanase action on this complex substrate, and likely each CE serves its own biological role. However, the synergistic effect observed between the CEs was rather subtle compared with the impact of *Dm*CE6A alone on xylanase hydrolysis. Acetylation of the xylan backbone likely contributes a greater hindrance to the action of a xylanase, while cleavage of feruloyl moieties may instead be important to loosen the cell wall matrix and improve accessibility for other hydrolytic enzymes by *e.g.*, breaking covalent bonds to lignin or between diferulate-linked GAX chains.

### Acetic acid and ferulic acid release from model and native substrate

To complement the studies of how the *D. mossii* CEs can facilitate sugar release from complex biomass and gain deeper insights into the enzymes’ roles in the process, we also monitored the release of acetic and ferulic acid. Acetic acid release was determined from the previously described enzyme reactions on corn cob biomass after 24 h. The highest amount of acetic acid was released when *Dm*CE6A was present, either alone or together with *Dm*CE1A and/or *Dm*CE1B, resulting in the release of 3.6 ± 0.1 mM of acetic acid. In reactions where *Dm*CE1B or *Dm*CE1B_nt was the only CE, 1.8 ± 0.1 mM of acetic acid was released, which might indicate that *Dm*CE1B_nt is a bifunctional acetyl-feruloyl esterase. No release of acetic acid could be observed for either *Dm*CE1B_ct or *Dm*CE1A.

Thin layer chromatography (TLC) was used to further investigate the enzymes’ ability to deacetylate a range of different sugars. Acetylated xylose represents the most ideal substrate for these assays, but unfortunately staining of this substrate even after several rounds of experimental optimization was poor and the results are therefore not easily interpreted. The method however worked well for a range of differently acetylated mannose substrates and fully acetylated glucose (Figs. S5–S7). These additional substrates enabled investigation of any preferences in sugar stereochemistry for the acetyl esterases. However, no such preference was observed, as each enzyme generated a specific reaction product profile that did not alter with respect to the substrates tested (Table S2). In agreement with the acetic acid release studies on biomass, there was no observable activity for either *Dm*CE1B_ct or *Dm*CE1A. *Dm*CE1B and *Dm*CE1B_nt were able to deacetylate all the substrates to mono- or diacetylated products. From these studies and limitations in available standards, it is not possible to conclusively determine which positions on the sugar rings remain untouched by the CE1 domains. The results however indicate that *Dm*CE1B and *Dm*CE1B_nt were capable of cleaving acetyl groups from the majority of sugar ring positions, and seemingly either acetylation of the *O*-2 and *O*-3 positions could not be hydrolyzed. Interestingly, xylan can only be acetylated on *O*-2 and *O*-3 in plant biomass, as *O*-1 and *O*-4 are linked to other xylose residues in the polymer. *Dm*CE6A was able to fully deacetylate all substrates, thus demonstrating promiscuous behavior toward acetyl groups on different positions and sugars.

In an attempt to mitigate the poor staining of acetylated xylose in TLC analyses, nuclear magnetic resonance (NMR) spectroscopy was used to further investigate the deacetylation pattern of *Dm*CE6A. 1,2,3,4-tetra-*O*-acetyl-d-xylopyranose (TetAcXyl) was used as a model substrate (Fig. S8). Due to the limited solubility of TetAcXyl in water, the reaction had to be followed in two different sets of experiments. Firstly, the decrease of TetAcXyl was monitored by sampling the reaction at different time intervals and dissolving the samples in dimethyl sulfoxide-d6 (DMSO-d6). Secondly, the increase of products obtained using enzymatic degradation was followed in D_2_O. The NMR results confirmed full degradation of TetAcXyl to xylose and acetic acid. Although intermediate species of partly deacetylated TetAcXyl could be observed in the experiments, these species were too short-lived and their concentrations were too low for determining their molecular structure (Figs. S9 and S10). This was likely due to increased solubility of the intermediates in D_2_O and a rapid conversion to xylose by *Dm*CE6A without substantial buildup of intermediates. Our other substrate, tri-*O*-acetyl-d-xylopyranosyl azide, suffers from similar solubility issues and was not pursued.

Ferulic acid (FA) release from corn cob biomass after 24 h of hydrolysis was studied using ultraperformance liquid chromatography–high-resolution mass spectrometry (UHPLC-HRMS). In the control reactions containing only Xyn11A, no FA release could be detected, nor was it detected in reactions supplemented with *Dm*CE1B_ct, *Dm*CE1A, or *Dm*CE6A. In reactions containing *Dm*CE1B or *Dm*CE1B_nt, FA was however detected, confirming the enzyme’s feruloyl esterase activity (Table S3). Curiously, an overlay of the LC chromatograms revealed a peak at 3.6 min in the analysis of the *Dm*CE1A-supplemented reaction (which resulted in a 237.3767 m/z in negative ionization mode). Tandem mass spectrometry was deployed in an effort to identify this chemical compound, and the MSMS fragmentation resulted in fragments at 145.0288, 119.0488, 117.0328, 163.0388 m/z, which did not result in any matches in publicly available databases when both the precursor and fragments ions were used as a query. Trimethoxycinnamic acid has a matching molecular weight (minus the mass of one hydrogen atom for running MS in negative mode) but the retention time of the unknown precursor did not match that of the standard molecule (4.4 min) nor the fragmentation pattern (103.0554, 133.0659, 115.0555, 118.0422, and 105.0711 m/z). We further utilized SIRIUS 4.5.0 software (43) with integrated Zodiac (44), CSI: FingerID (45), and CANOPUS (46, 47) tools, which collectively suggested that the unknown precursor might be either *p*-coumaric acid ethyl ester, C_11_H_12_O_3_, in a [M+HCOO]- form with six out of 12 experimental fragment peaks being explained by computational fragmentation trees (48) in SIRIUS or 2,3-dihydropropyl (E)-3-(4-hydroxyohemyl)prop-2-enoate (*p*-coumaric 2,3-dihydroxypropyl ester), C_12_H_14_O_5_, in its deprotonated form, with ten out of 12 experimental peaks being explained (Table S4, Fig. S11). Both compounds belong to the coumaric acid esters class according to CANOPUS (46, 47). Similar main fragments have also been reported for *p*-coumaroyl-1-5-quinidie lactone (49), which further supports the class identity as a coumaric acid ester. The exact identity of the unknown compound remains unresolved, but it is likely a type of coumaric acid ester that may represent a previously unidentified noncarbohydrate decoration of GAX. Future work using preparative HPLC and structure elucidation by NMR would be needed for absolute identification of the molecule and could both inform on the activity of *Dm*CE1A and reveal new structural motifs in xylan.

### Structural determination of Dm*CE1B_ct*

#### Overall structure

**Figure 3:**
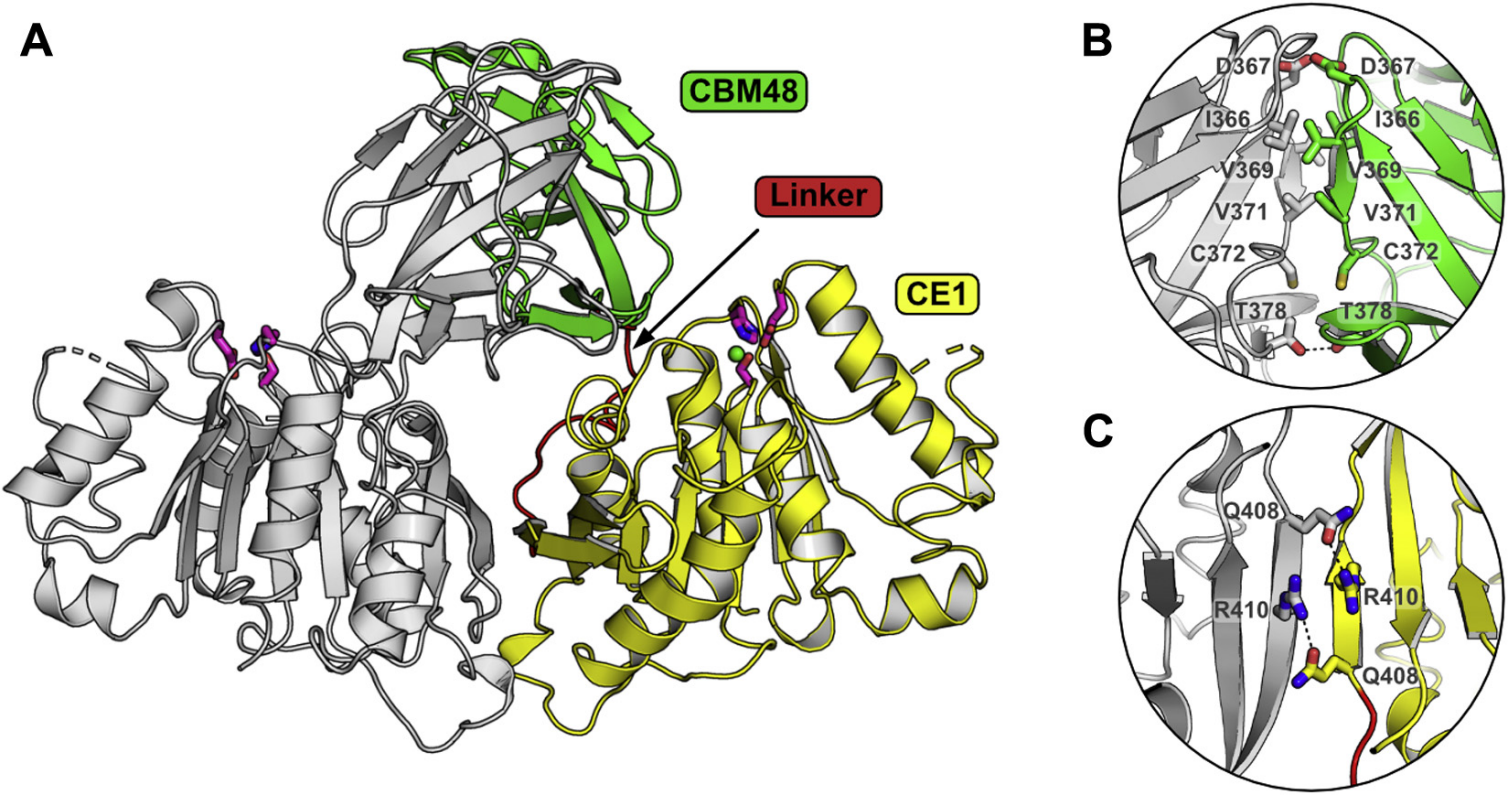
**Overall structure of *Dm*CE1B_ct.***A*, the overall structure of the proposed dimeric biological unit of *Dm*CE1B_ct with one monomer shown in *gray* and the other colored with the CBM48 in *green*, linker region in *red*, and the CE1 domain in *yellow*. The Glu-His-Ser residues of the catalytic triad are colored in *magenta* and the chloride ion found in the active site of one of the protomers is shown as a *green sphere*. Few interactions are observed at the dimer interface and the prominent interactions across the CBM48 (*B*) and CE1 (*C*) domains are shown.

Despite the biochemical studies of *Dm*CE1B_ct, its activity and substrate specificity remain elusive. To gain greater insights into the enigmatic activity of *Dm*CE1B_ct, we pursued its structural determination by macromolecular crystallography. We were able to obtain crystals and solve the structure using molecular replacement with the putative acetyl xylan esterase from *Bacteroides intestinalis* (PDB: 6NE9) as the template. *Dm*CE1B_ct forms two discrete globular domains, a ten-stranded *β*-sandwich domain consistent with the CBM48 family structure (residues 294–393) connected *via* a short linker (residues 394–404) to a three-layer *αβα*-sandwich (residues 405–656) typical of CE1 enzymes. Two protein molecules were present in the asymmetric unit and aligned along a noncrystallographic symmetry operator with the two *α*/*β*-hydrolase domains packed with *β*1 of their *β*-sheets adjacent and antiparallel to each other and the CBM48 domains clasping each other (Fig. 3*A*). The interaction in the dimer masks 10% of accessible surface, as determined by PISA (50), but with only a few key interactions (Fig. 3, *B* and *C*) suggesting the interaction might be biologically relevant. Molecular weight analysis by size-exclusion chromatography suggests dimeric oligomerization in solution of the full-length *Dm*CE1B and *Dm*CE1B_ct (Fig. S12). The two protomers showed high structural similarity to each other, with an all-atom root mean square deviation of 1.01 Å. Two stretches of residues in the *α*/*β*-hydrolase domain (residues 493–508 and 572–574) were unable to be modeled in both protomers likely owing to flexibility in these regions.

#### Comparison to other CE1 enzymes

A search for structurally homologous proteins using DALI revealed several *α*/*β*-hydrolases as homologs, but only four shared a similar multidomain CBM48-CE1 structure: the *B. intestinalis* enzymes Bacint_01039 (putative feruloyl esterase; PDB: 6NE9; seq. id. 52%), Bacint_01033 (putative acetyl xylan esterase; PDB: 6MOU and 6MOT; seq. id. 51%), wtsFae1A, and wtsFae1B (PDB: 6RZO and 6RZN; seq. id. 52% and 38%, respectively, (51)). Although only sharing 38 to 52% sequence identity, the structures of all these multidomain enzymes are closely related (root mean square deviation of C*α* atoms 1.5–1.8 Å) with the same domain positioning and dimer interface (Fig. S13).

In *Dm*CE1B_ct, the positioning of the CBM48 domain relative to the CE1 domain is stabilized by hydrogen bonds through several residues: the main chain amide of Ser383 to the main chain carbonyl of Gly451, the hydroxyl of Tyr361 with an oxygen of the carboxylate of Glu452, the N^δ^ of His360 with both O^ε1^ and O^ε2^ of the carboxylate from Glu639, and a salt bridge between Glu390 and Arg644 (Fig. S14*A*). Further, the linker connecting the domains in *Dm*CE1B_ct is packed along one face of the CE1 domain and its position is also stabilized by several hydrogen bonds: the carboxylate of Asp468 with the main chain amide of Gly407 and N^δ^ of His406, the N^η^ of Asn469 with main chain carbonyls of Tyr398 and Asn400, and an N^η^ of Arg645 with an oxygen of the carboxylate of Glu393 (Fig. S14*B*). Most of the hydrogen bond networks observed in the *Dm*CE1B_ct between and linking the domains are conserved in the other structurally determined CBM48-CE1 enzymes, except for wtsFae1B, which contains a significantly different linker region leading to different interactions at the interface between its domains. From the few CE1 structures determined to date, the subset of the family that contains CBM48 domains appears to maintain a very similar overall domain organization and oligomerization properties.

**Figure 4:**
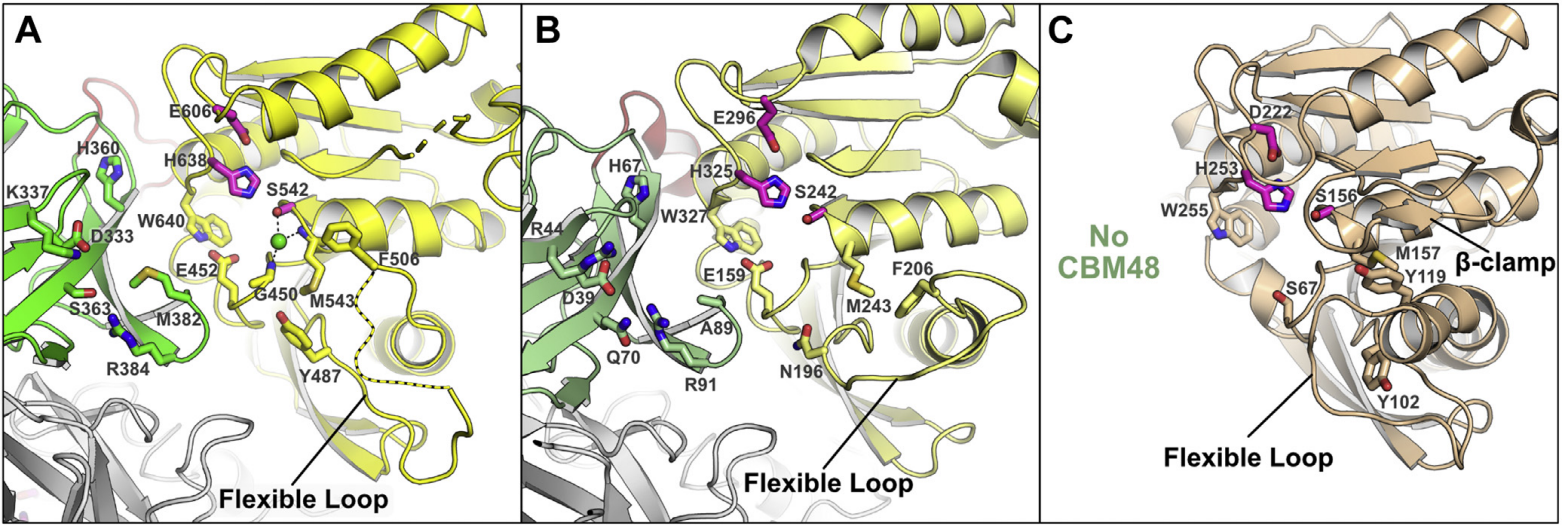
**Comparison of the active site of *Dm*CE1B_ct with CE1 members.***A*, the active site of *Dm*CE1B_ct colored as in Figure 3 with one monomer shown in *gray* and the other colored with the CBM48 domain in *green*, linker region in *red*, and the CE1 domain in *yellow*. Residues lining the cleft between the CBM48 and the CE1 domains are shown and the location of the flexible loop region is highlighted. The active sites of (*B*) wtsFae1A (PDB: 6RZO) and (*C*) *Am*Fae1A from *A. mucronatus* (PDB: 5CXX) shown using the same orientation. The flexible loop and *β*-clamp regions are highlighted.

Comparison to other CE1 family members enables annotation of the catalytic triad in *Dm*CE1B_ct (Ser542, His638, Glu606), which is found in a groove between the CE1 and CBM48 domains (Fig. 4*A*). A chloride ion was observed in the active site of the protein coordinating with the hydroxyl of the catalytic serine and the main chain amides of Gly450 and Met543, which likely comprise the oxyanion hole. No inhibition of *Dm*CE1B_ct-mediated hydrolysis of 4-MU-Ac was observed in chloride concentrations up to 100 mM, indicating that the presence of the ion is likely an artifact of crystallization. Sequence similarity in the groove immediately surrounding the catalytic triad is high among the close CE1 family homologs and suggests a common mechanism and substrate binding mode present in the *Dm*CE1B_ct. Two distinguishing active site features have been defined in the CE1 family. The first is the presence or absence of a long flexible loop between *β*2 and *α*2 of the *α/β*-hydrolase fold, such as in wtsFae1A and wtsFae1B (PDB: 6RZO and 6RZN, respectively), where this loop wraps around one end of the active site and has been proposed to provide substrate specificity to the enzyme (51) (Fig. 4*B*). The second is the presence or absence of a *β*-clamp or helical region between *β*4 and *α*5 of the *α*/*β*-hydrolase fold, such as in *Am*Fae1A from *A. mucronatus* (PDB: 5CXX; (52)) and Fae1A from *B. intestinalis* (PDB: 5VOL), which can aid in building up and capping the active site cleft (53) (Fig. 4*C*). While two residues could not be modeled in the corresponding *β*-clamp or helical region in *Dm*CE1B_ct, the region forms a short loop that does not comprise either motif and does not protrude toward the binding cleft. *Dm*CE1B_ct has the flexible loop feature, though ten of the residues in the region could not be fully resolved in the final model due to a lack of electron density. While there is significant sequence diversity in this loop region among the homologs where it is found, it is likely that the flexible loop in *Dm*CE1B_ct is positioned close to the cleft and possibly aids in substrate specificity in a similar way to that proposed for wtsFae1-A (51).

The *Dm*CE1B_CBM48 domain is most closely related to the analogous domains of the previously mentioned CBM48-CE1 members (PDB: 6NE9, 6MOU, 6MOT, 6RZO and 6RZN; 42–47% sequence identity). While all these domains are similar to the canonical starch binding CBM48 members, the previous work on wtsFae1A and wtsFae1B showed that the domains lacked starch binding properties and proposed a subfamily within CBM48 for domains appended CE1 domains (51). Interestingly, the PULDB lists plenty of CE1 domains fused to CBM48 domains, but not to other CBM family members (14). In *Dm*CE1B_ct and its close multidomain homologs, the CBM48 packs against the CE1 domain with a face of one of its *β*-sheets toward the active site (Fig. 4*A*). While this face does not contain a large amount of hydrophilic or aromatic residues, whose functionalities are commonly associated with carbohydrate binding sites, four residues on the face of the *Dm*CE1B_ct *β*-sheet (Asp333, Ser363, Lys337, and Arg384) are either conserved or functionally conserved among the close homologs. Molecular docking studies of wtsFae1A with an arabinoxylan fragment ester linked to ferulate previously identified this region as a likely location for xylan binding. To test the binding abilities of the CBM48 domain, *Dm*CE1B_CBM48 (14.7 kDa) and *Dm*CE1A_CBM48 (14.4 kDa) were heterologously produced in *E. coli*. The two proteins did not bind to the insoluble polysaccharides ivory nut mannan, cellulose, birch xylan, beech xylan, barley *β*-glucan, and potato starch, nor to the water-soluble polysaccharides carboxymethyl cellulose, galactomannan, glucomannan, wheat arabinoxylan, and xyloglucan (data not shown). *Dm*CE1B_CBM48 and *Dm*CE1A_CBM48 may still help direct the catalytic domains to substrates in the more complex plant cell wall. Possibly, in the case of *Dm*CE1B, the CBM domain might simply act as a spacer between the catalytic domains.

#### Complex of *Dm*CE1B_ct with methyl ferulate

To investigate potential ligand binding in *Dm*CE1B_ct, we pursued a protein–ligand complex by soaking crystals with methyl ferulate (MFA). We determined the structure of the protein in a different crystal form, which yielded the same dimer interface observed in the apo structure, except for the addition of a partially occupied disulfide bond observed between the two Cys372 residues of each protomer along the noncrystallographic symmetry operator. This disulfide bridge was absent in the structure of the apo protein, which was crystallized at a lower pH (pH 5 *versus* pH 8 for the complex) but may be biologically relevant to stabilize the dimer formation. Two MFA molecules were observed and modeled (Fig. S15*A*): one at the interface between the two CE1 domains of the dimer (Fig. S15*B*), and the other close to the active site in one of the protomers (Fig. S15*C*).

**Figure 5:**
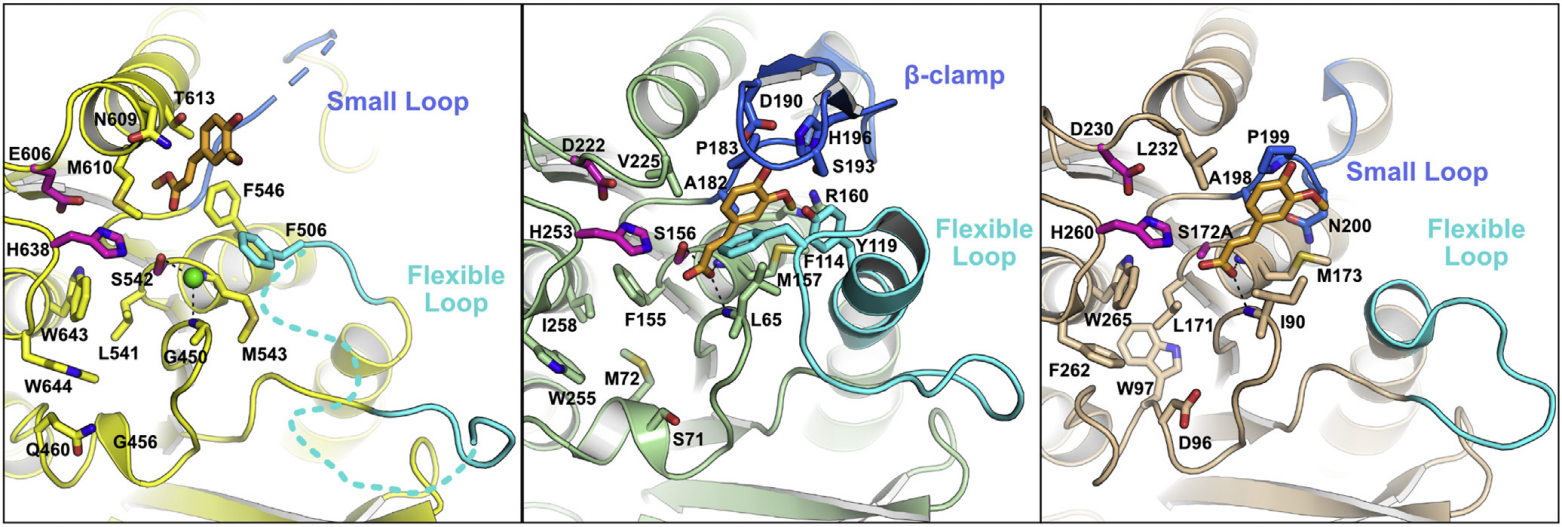
**Comparison of aromatic ligand binding of *Dm*CE1B_ct and CE1 members.***A*, the active sites of *Dm*CE1B_ct in complex with MFA, (*B*) *Am*Fae1A from *A. mucronatus* in complex with FA (PDB: 5CXX), and (*C*) the S172A variant of *Ct*Fae from *C. thermocellum* in complex with FA (PDB: 1JT2). The residues of the catalytic triad are colored in *magenta* and the chloride ion in *Dm*CE1B_ct is shown as a *green sphere*. The aromatic ligands are shown as *orange sticks* and the regions corresponding to the flexible loop and *β*-clamp/helical regions are colored in *cyan* and *navy blue*, respectively. Note that the smaller and further setback loop present in *Dm*CE1B_ct in the *β*-clamp/helical region likely contributes to the positioning of MFA further from the catalytic serine.

The MFA bound between the two protomers, which is far from either active site and makes minimal interactions with protein, is likely an artifact and not of biological relevance. The second MFA molecule is found on the edge of the active site groove positioned with its methyl ester ∼5 Å from the proposed catalytic center between the catalytic serine and the oxyanion hole (Fig. 5*A*). The MFA in *Dm*CE1B_ct makes no direct electrostatic interactions with the protein and is not positioned in line for catalytic cleavage. This is not greatly surprising since the enzyme lacked appreciable activity on MFA. MFA is positioned in proximity to where FA has been observed in structures of the *A. mucronatus Am*Fae1A (PDB: 5CXX; (52)) and *Clostridium thermocellum Ct*Fae (PDB: 1JT2; (54)) though the positioning of the ligands in the latter structures is in line to facilitate catalysis (Fig. 5, *B* and *C*). In the *Am*Fae1A FA complex, positioning of the ligand is facilitated by significant interactions from the *β*-clamp and an extended *α*2 occupying a similar position as the flexible loop region in other homologs (52). Interestingly, while *Ct*Fae lacks a large flexible loop region, an equivalent *β*-clamp or helical region, and thus the interactions supported by those regions, the FA molecule is still positioned in the binding cleft in the same position as in *Am*Fae1A (52, 54). In the *Ct*Fae FA complex, the positioning of the ferulic acid ligand is facilitated primarily by packing of the face of the aromatic ligand against the face of a proline residue in a small loop from the region equivalent to the *β*-clamp or helical region (52). In *Dm*CE1B_ct, the positioning of MFA further away from the catalytic center is possibly due to the smaller loop corresponding to the *β*-clamp or helical region. This may suggest that the substrate for the enzyme is in fact a larger molecule, which would fit into the larger cleft created by the small loop in the *β*-clamp/helical region.

## Discussion

The human gut is a highly competitive environment with extreme densities of microorganisms. A large part of the nutrients in the distal gut is dietary fiber, where xylan polysaccharides are a major component of ingested plant cell walls that cannot directly be metabolized by the host enzymes. The ability to remove noncarbohydrate groups from polysaccharides is crucial for the gut microbiota as it enables them to efficiently access the underlying sugars. For complex xylans, both acetyl and feruloyl groups need to be addressed. Acetyl xylan esterase activity enables access to the xylan main chain for *endo*-acting xylanases, and the activity is common in multiple CAZy families (CE1-7 and 12) (15). Feruloyl esterase activity is on the other hand only reported in CE1. Feruloyl esterases may support biomass degradation in less direct ways, such as by removing single feruloyl groups, which would give access to underlying carbohydrates, but also to help loosen the cell wall matrix by cleaving covalent links between feruloyl groups and lignin, as well as diferulate cross-links between separate xylan chains. Lignin is not believed to be a major nutrient source in the gut, though it may be modified by the microbiota (55). The separation of lignin and carbohydrates through the action of feruloyl esterases likely has strong implications for nutrient acquisition for individual microorganisms such as *D. mossii*, but also in the structure of the lignin particles themselves, which may be more prone to aggregation after release from hydrophilic glycans.

PULs are efficient systems that have been argued to give Bacteroidetes bacteria a competitive edge in nutrient scavenging, whether the organisms reside in the gut or in other environments (18, 56), and the importance of both acetyl xylan esterase and feruloyl esterase activities are mirrored by the number of PULs encoding the corresponding CE families (14). Multicatalytic CEs, *i.e.*, comprising two or more CE domains, have to our knowledge only been characterized in our own recent work where two different enzymes composed of CE6-CE1 fusions were investigated (32). Enzymes comprising dual CE1 domain setups are found in over 50 PULDB entries from a range of Bacteroidetes species, indicating an important role in biomass turnover and this work represents the first study of these types of enzymes. Screening the PULDB also revealed nine multicatalytic CE6-CE1-CE1 enzymes, most of which can be found in predicted PULs from *Flavobacterium* sp. though none of them have yet been characterized. Interestingly, all of these CE6-CE1-CE1 enzymes possess a predicted CBM48 domain, and they likely act in synergy in a similar manner to the enzymes studied here and previously studied CE6-CE1 fusions (32).

Our study of the multiple CEs found in PUL 17 of *D. mossii* shows that each PUL-encoded CE domain is unique, where *Dm*CE6A is clearly the dominant acetyl xylan esterase, able to rapidly deacetylate all carbohydrates tested as well as release acetate from complex biomass. Of the CE1 domains, *Dm*CE1B can be ascribed as the feruloyl esterase of the PUL, where the N-terminal domain was active both on model substrates and able to release ferulic acid from complex GAX-rich biomass. Our results also suggest that *Dm*CE1B_nt may be a bifunctional feruloyl esterase/acetyl xylan esterase, as it was further able to deacetylate a range of carbohydrates as well as release acetate from complex biomass. Interestingly, *Dm*CE1A and *Dm*CE1B were more active on MSA than on MFA, though MFA is generally the model feruloyl esterase substrate with the highest reported activities (32, 57, 58). However, the activities on MSA were comparably low and the enzymes might perform better on substrates more closely resembling the structures found in the plant cell wall, such as 5-*O*-*trans*-feruloyl-l-arabinofuranose (57, 59), though these are not commercially available.

The structures targeted by *Dm*CE1A and *Dm*CE1B_ct in the plant cell wall matrix remain elusive, though we could still observe the ability of each CE1 enzyme to boost the action of a xylanase in various combinations. Possibly, these domains are acting on specific structures of the biomass that have remained difficult to identify and study due to general polysaccharide extraction protocols using strong base that may cleave ester bonds prior to chemical/structural analyses, as well as low abundances complicating chemical analyses (60). The incorporation of multiple CEs into a single PUL however strongly suggests that these are all biologically important for *D. mossii* and have different roles to help the bacterium compete for nutrients in the human gut. By solving the structure of *Dm*CE1B_ct, including both the CBM48 and CE1 domains, we provide a basis for future studies to unravel the true substrate of this domain and its interaction with the bifunctional N-terminal CE1 domain. It is currently unknown how flexible the two CE1 domains are with respect to each other, but likely this greatly affects the properties of the full-length enzyme during catalysis.

The interplay between the members of the gut microbiota and their respective enzymes is only beginning to be unraveled. Similarly, the fate of lignin in the human gut is poorly investigated, but enzymes such as feruloyl esterases likely have key roles in releasing it from covalently attached carbohydrates, thus affecting both its chemical and physical properties, notably its hydrophobicity. Lignin itself may possibly also have health-promoting properties. For example, lignin extracted from the deciduous tree *Acacia nilotica* was reported to possess antihyperglycemic properties (61, 62, 63). However, lignin is highly heterogeneous (62) and its influence on the microbial gut and the host’s health likely depends on a range of factors such as the type of lignins entering the gut and the individual’s microbial community composition. Whether the enzymes encoded by PUL 17 of *D. mossii* give it a competitive advantage in the environment, in a so-called selfish manner (17), or if they support the greater microbial community by enabling cross-feeding of multiple organisms is not known. Either way, the existence of such enzymes likely enables a more efficient metabolism of dietary fiber, thus fueling SCFA production, which is strongly correlated to overall gut health. *D. mossii* has not received much attention in the literature, especially with regard to its capabilities in metabolizing complex glycans, but this work represents a starting point to understand its importance in the human gut and possible implications for improved human health.

## Experimental procedures

All chemicals were purchased from Sigma-Aldrich if not stated otherwise.

### Cloning of CEs from *D. mossii*

Genomic DNA of *D. mossii* was purchased from DSMZ (DSM 22836). Phusion High-Fidelity DNA Polymerase (Thermo Fisher Scientific) was used to amplify the CE genes by PCR (excluding signal peptide sequences that were identified using SignalP; (36)). The cloning of *Dm*CE1B_nt and *Dm*CE1B_ct included the CBM48 domain, as in our previous work we found the presence of such domains to be vital for enzyme stability (32). Primer pairs (Table S5) for the CE constructs included overhangs homologous to the targeted cloning site in the vector pET28a-TEVc (generously provided by Dr N. Koropatkin, University of Michigan), which was digested with *Nde*I and *Xho*I (Thermo Fisher Scientific) prior to fusion with the In-Fusion HD cloning kit (Takara Bio). Chemically competent *E. coli* stellar cells (Takara Bio) were transformed with the fusion mix. Transformants were selected on LB (lysogeny broth) agar plates, containing 2% w/v agar and 50 μg/ml kanamycin, and incubated over night at 37 °C. Single colonies were subcultured in 5 ml LB + 50 μg/ml kanamycin and their plasmids extracted (GeneJET Plasmid Miniprep kit; Thermo Fisher Scientific), which were then transformed into chemically competent *E. coli* BL21(DE3) (Sigma-Aldrich). Plasmid construction was verified by sequencing (Eurofins genomics).

### Production and purification of CEs

All proteins were produced in 1 l cell culture batches. After reaching mid-log phase (OD 0.4–0.6; incubation at 37 °C and 180 rpm) protein production was induced by addition of 0.2 mM isopropyl-*β*-d-1-thiogalactopyranoside (IPTG) and proteins were expressed during overnight cultivations at 16 °C. The cells were harvested by centrifugation followed by disruption using sonication. Cell debris was separated from the crude lysate by centrifugation (30 min at 4000*g*). Purification of the recombinant enzymes was accomplished by immobilized metal ion affinity chromatography (IMAC) by methods similarly described in Bååth *et al.* (64). The purified proteins were buffer exchanged into IMAC binding buffer (50 mM tris(hydroxymethyl)aminomethane pH 8.0, 250 mM NaCl, 5% glycerol) and imidazole concentrations were reduced to <1 mM using 10 kDa cutoff centrifugal filter units (Amicon Ultra 15, Merck-Millipore).

### Biochemical characterization

The molecular weight and purity of the proteins were assessed by sodium dodecyl sulfate–polyacrylamide gel electrophoresis (SDS-PAGE) using Mini-PROTEAN TGX Stain-Free Gels (BIO-RAD). Extinction coefficients and molecular weights of the proteins were estimated using the software Benchling. These values were used together with a Nanodrop 2000 Spectrophotometer (Thermo Fisher Scientific) to assess protein concentrations.

### Assays on model substrates

The enzymes were biochemically characterized at 37 °C on model substrates as described in Kmezik *et al.* (32). pH optima were determined using 1 mM 4-MU-Ac (Fig. S4). Kinetic parameters were determined using 4-MU-Ac (0.01–5 mM), *p*NP-Ac (0.1–10 mM), MSA (0.01–0.4 mM), MFA (0.01–0.4 mM), MCA (0.01–0.4 mM), and M*p*CA (0.01–0.4 mM). The data were fitted to the Michaelis–Menten equation using the software OriginPro 2018. For reactions that could not be saturated with substrate, *k*_*cat*_*/K*_*M*_ was estimated using linear regression. *Dm*CE1B_ct was additionally tested on *p*NP-butyrate (3 mM), *p*NP-octanoate (3 mM), *p*NP-dodecanoate (6 mM), and *p*NP-palmitate (3 mM).

### Boosting studies with xylanase on corn cob biomass

The ability of the *D. mossii* enzymes to boost the activity of the commercially available *endo*-1,4-*β*-xylanase Xyn11A (CAS 9025-57-4; Megazyme) was determined on 5% w/v ball milled and freeze-dried corn cob biomass. The reactions were incubated at 1000 rpm and 37 °C in a thermomixer (Eppendorf). The amount of reducing sugars was determined using 3,5-dinitrosalicylic acid (DNSA; (65)) as described in Kmezik *et al.* (32). Xylose was used as standard to translate absorbance values at 575 nm into reducing sugar equivalent concentrations. The xylooligosaccharide product profiles were determined by measuring X_1_, X_2_, X_3_, X_4_, X_5_, and X_6_ concentrations using HPAEC-PAD (high-performance anion-exchange chromatography with pulsed amperometric detection) on a Dionex ICS-5000+ (Thermo Fisher Scientific) equipped with a Dionex CarboPac PA200 column (Thermo Fisher Scientific) (66).

### Binding studies

Binding studies to assess the ability of *Dm*CE1A_CBM48 and *Dm*CE1B_CBM48 to bind insoluble polysaccharides were performed using pull-down studies as described in Kmezik *et al.* (32), except 50 mM sodium phosphate buffer (pH 6.5) was used as the buffer, on the insoluble polysaccharides: ivory nut mannan (Carbosynth), cellulose (Merck), birch xylan (Merck), beech xylan (Merck), mixed-linkage barley glucan (Megazyme), and potato starch (Merck). All polysaccharides were washed three times in the aforementioned buffer before being used in the assay. Binding studies using soluble polysaccharides by affinity gel electrophoresis were performed as previously described (67, 68) using carboxymethylcellulose, galactomannan (Megazyme), glucomannan (Megazyme), wheat arabinoxylan (Megazyme), and xyloglucan (Megazyme). All polysaccharides were used at a concentration of 0.5% w/v in the polyacrylamide gels.

### Thin-layer chromatography

An aluminum-backed silica TLC plate (Silicagel 20 x 20, VWR) was cut to the desired size and a pencil line was drawn 1 cm from the bottom of the plate. Standards were prepared at a concentration of ∼20 mM, in some cases with 5% DMSO to aid solubilization. Samples and standards were spotted in 1 μl volumes, separated by 8 mm. Enzyme reactions were carried out over 4 h and with substrate at 5 mM; these samples were spotted on plates five times in the same position to allow sufficient staining. Running buffer (toluene:acetone 4:1) was poured into a glass chromatography tank to a height of less than 1 cm. The TLC plate was placed into the tank, and samples were allowed to migrate until the buffer reached the top of the plate. The plate was removed and air-dried. The plate was then dipped in a staining solution of 8% H_2_SO_4_ in ethanol. After air-drying, the plate was incubated at 120 °C until spots appeared, typically after 5 min.

### Acetic acid and ferulic acid release

Release of acetic acid was quantified for samples containing 5% corn cob using the acetic acid (rapid manual format) assay kit K-ACETRM (Megazyme) according to the supplier’s instructions. Release of ferulic acid was determined using UHPLC-HRMS on a 1290 Infinity II LC system with an Acquity UPLC HSS T3 column coupled to a 6550i funnel QTOF. A methanol gradient was used for separation and the QTOF was calibrated on a mass range of 100 to 3200 m/z. The monoisotopic mass and the expected ions of the compound were calculated based on the given molecular formula: C_10_H_10_O_4_.

### Nuclear magnetic resonance

All NMR measurements were conducted on a Varian Inova 500 MHz operating at 11.7 T with a 5 mm HFX-probe. The ^1^H-spectrum parameters included an 8 ms ^1^H-detection pulse, 2 s acquisition time, and 5 s recycle delay. ^13^C-spectra were acquired using a 14 ms 13C-detection pulse, 1 s acquisition time, and 5 s recycle delay. For structure elucidation, several standard experiments were used. Correlation spectroscopy (COSY) was recorded using 2048 scans with 256 increments. Heteronuclear single quantum coherence spectroscopy (HSQC) was recorded using 64 scans with 384 increments and a transfer delay of 3.425 ms corresponding to a 145 Hz J-coupling. Heteronuclear multiple bond correlation (HMBC) was recorded using 64 scans with 512 increments and a transfer delay of 62.5 ms, corresponding to an 8 Hz J-coupling. For all samples, the DMSO signal was used as chemical shift reference.

### Mass spectrometry

Verification of ferulic acid in the samples was performed on an Agilent 6550 high-resolution mass spectrometry (HRMS) coupled to an Agilent Infinity 1290 II UHPLC system. Metabolites were separated on an UPLC HSS T3 (1.8 μm, 2.1 × 100 mm, Waters) column with a water-MeOH gradient solvent system containing 0.04% formic acid. The gradient started at 5% MeOH with formic acid (MPB) and ramped to 100% MPB over 6 min and held for 4.50 min at 100% MPB. Column temperature was set to 45 °C and the flow at 0.4 ml/min. Mass spectra were acquired using a Jetstream ESI source in negative ionization mode scanning from 50 to 1700 m/z at 1.67 spectra/s. The capillary voltage was set at 3500 V. The source parameters were as follows: gas temperature at 175 °C, drying gas flow at 12 l/min, nebulizer at 45 psig, sheath gas temperature at 375 °C, and flow at 11 l/min and fragmentor at 300 V. Ferulic acid was identified based on its accurate mass (193.4546 m/z) and retention time (2.8 min). MS/MS data was acquired for the selected precursor at 237.3767 m/z identified in reactions containing *Dm*CE1A and for a standard of 3,4,5-trimethoxycinnamic acid (molecular weight 238.24 g/mol) by using three different collision energies (10, 20 and 40 eV) with a duty cycle of 2.032 s/cycle. MS data was acquired with MassHunter Workstation Data Acquisition.

### Structure determination of *Dm*CE1B_ct

Crystallization conditions were screened with a Mosquito robot (SPT Labtech) using the JCSG+ screening kit (Molecular Dimensions) in MRC sitting drop plates. Protein was dialyzed into 50 mM Tris buffer at pH 8.0 containing 50 mM NaCl prior to screening and screens were setup with a reservoir volume of 40 μl and protein mixed with reservoir solution in a 1:1 ratio in 0.6 μl drops. Within a month, crystals of varying quality were observed in several of the conditions in the screen and a crystal obtained from the condition containing 0.1 M citrate pH 5 and 20% w/v PEG 6000 was utilized for X-ray diffraction data collection. The crystal was mounted and flash frozen in liquid nitrogen in the absence of additional cryo protectant. Crystals grown in an optimized condition containing 0.1 M Tris pH 8 and 25% w/v PEG 6000 were utilized for soaking of the MFA ligand and were soaked in reservoir solution containing a saturating amount of MFA for 1 min prior to flash freezing in liquid nitrogen. A data set of the apo protein diffracting to 1.69 Å was collected at the BioMAX beamline at MAX IV Laboratory (March 27th, 2020). The data set was processed by XDS (69) and the structure was determined by molecular replacement with Auto-Rickshaw (70, 71), with MoRDa (72) identifying and using PDB accession 6NE9, a putative acetyl xylan esterase from *B. intestinalis* (BACINT_01039), as the search template. An initial model was built using autobuilding in ARP/wARP (73, 74, 75, 76). A data set of the soaked crystal diffracting to 1.41 Å was collected at the BioMAX beamline at MAX IV Laboratory (June 11th, 2020). The data set was processed by XDS (69) and the structure determined by molecular replacement with Phaser (77) in Phenix (78) using the apo protein as the template. The MFA compound was created in Pymol and its restraints created with Phenix eLBOW (79). For both structures, Coot (80) and Phenix Refine (81) were used in iterative cycles of real space and reciprocal space refinement. The data collection, processing, and refinement statistics for both data sets can be found in Table S6.

## Data availability

The structures presented in this work have all been deposited in the Protein Data Bank (PDB) with the following codes: 7B5V and 7B6B. All remaining data are contained within the article.

## Supporting information

This article contains supporting information.

## Conflict of interest

The authors declare no conflicts of interest regarding this article.
