# Supplementary material for: A polysaccharide utilization locus from the gut bacterium Dysgonomonas mossii encodes functionally distinct carbohydrate esterases
Source: J Biol Chem. 2021 Mar 2;296:100500. doi: 10.1016/j.jbc.2021.100500 (PMC8040265; doi:10.1016/j.jbc.2021.100500)
Supplement: Supplemental Figures S1–S15 and Tables S1–S6 [file mmc1.pdf]

Supplemental information

A polysaccharide utilization locus from the gut bacterium *Dysgonomonas mossii* encodes functionally distinct carbohydrate esterases

Cathleen Kmezik, Scott Mazurkewich, Tomke Meents, Lauren Sara McKee, Alexander Idström, Marina Armeni, Otto Savolainen, Gisela Brändén, Johan Larsbrink

Table S1. Comparative amino acid sequence analysis against the most similar sequences for each enzyme compared to entries in the Protein Data Bank.

Enzyme	Closest homolog in PDB	Sequence identity [%] (query coverage [%])
*Dm*CE1A	feruloyl esterase of Bacteroides intestinalis (PDB accesssion: 6MOT)	53 (98)
*Dm*CE1B_nt	feruloyl esterase domain of the cellulosomal xylanase Z of *Clostridium thermocellum* (PDB accession: 1JJF)	50 (66)
*Dm*CE1B_ct	feruloyl esterase of Bacteroides intestinalis (PDB accesssion: 6MOT)	50 (96)
*Dm*CE1A_CBM48	acetyl xylan esterase of *Bacteroides intestinalis* (PDB accesssion: 6NE9)	43 (97)
*Dm*CE1B_CBM48	acetyl xylan esterase of *Bacteroides intestinalis* (PDB accesssion: 6NE9)	48 (91)

Table S2. TLC results for the carbohydrate esterases of PUL 17 of *D. mossii*. Shown are the acetylation patterns of the products for each substrate and enzyme combination.

<table>
<tr><th></th><th>DmCE1A</th><th>DmCE1B</th><th>DmCE1B_nt</th><th>DmCE1B_ct</th><th>DmCE6A</th></tr>
<tr><td>2,3,4-tri-Ac-β-Xylp-azide</td><td rowspan="7">No reaction</td><td rowspan="6">Mono- and di-acetylated sugar</td><td rowspan="6">Mono- and di-acetylated sugar</td><td rowspan="7">No reaction</td><td rowspan="7">Mono-saccharide</td></tr>
<tr><td>penta-Ac-β-Glcp</td></tr>
<tr><td>penta-Ac-β-Manp</td></tr>
<tr><td>2,3,4,6-tetra-Ac-β-Manp</td></tr>
<tr><td>Me-2,3,4,6-tetra-Ac-α-Manp</td></tr>
<tr><td>2,3,6-tri-Ac-α-Manp</td></tr>
<tr><td>Me-2,3-di-Ac-α-Manp</td><td>Mono-acetylated sugar</td><td>Mono-acetylated sugar</td></tr>
</table>

Table S3. Ferulic acid release from corn cob biomass after 24 h reactions with Xyn11A and different *D. mossii* CEs. The data was acquired using UHPLC-HRMS. For samples in which ferulic acid was present the detected mass to charge ratio is given, as well as the relative mass error to the theoretical mass as parts per million.

Enzyme	Expected m/z	Detected m/z	Error (ppm)
*Dm*CE1A	193.0506	-	-
*Dm*CE1B	193.0506	193.0505	0.5
*Dm*CE1B_nt	193.0506	193.0491	7.8
*Dm*CE1B_ct	193.0506	-	-
*Dm*CE6A	193.0506	-	-

Table S4: Computationally explained fragment peaks of the experimental data of precursor 237.0766. Possible matches to *p*-coumaric ethyl ester and 2,3-dihydropropyl (E)-3-(4-hydroxyohemyl)prop-2-enoate are based on fragmentation trees from SIRIUS 4.5.0 (1, 2).

Compound	Explained peaks (Da)	Formulas	Relative intensity
***p*-coumaric ethyl ester**	237.0766	C11H12O3	0.4037
	163.0387	C8H6O	0.0660
	145.0289	C8H4	1.000
	91.0384	C2H6O	0.0114
	89.0233	C2H4O	0.0276
	71.0139	C2H2	0.0092
	59.0133	CH2	0.0729
**2,3-dihydropropyl (E)-3-(4-hydroxyohemyl)prop-2-enoate**	237.0766	C12H14O5	0.4037
	163.0387	C9H8O3	0.0660
	149.0955	C10H14O	0.0063
	145.0289	C9H6O2	1.0000
	119.0493	C8H8O	0.1664
	119.0415	C8H7O	0.0418
	117.0337	C8H6O	0.20242
	91.0384	C3H8O3	0.0114
	89.0233	C3H6O3	0.0276
	71.0139	C3H4O2	0.0092
	59.0133	C2H4O2	0.0729

Table S5. Primers used in this study to amplify *D. mossii* genes. Primer pairs for each construct consisting of a forward primer (F) and a reverse primer (R) are written from 5’ to 3’ end. Overhangs homolog to the used cloning site in pET28a-TEVc are written in bold and underlined

Construct		DNA sequence
*Dm*CE1A	F	**CTTCCAGGGCCATAGT**CAAGACCTAGAGGGAAGTATTTCGTC
	R	**TGGTGGTGCTCGAGTCTA**CTAATTTTGTTTGAATATTAGAGGTAAAAATTGATTAAG
*Dm*CE1A_CBM48	F	**CTTCCAGGGCCATAGT**CAAGACCTAGAGGGAAGTATTTCGTC
	R	**TGGTGGTGCTCGAGTCTA**CGCCATCCTTCCACAACC
*Dm*CE1B	F	**CTTCCAGGGCCATAGT**ATCAGTCAAAATTATAAAAGTGTGCCG
	R	**TGGTGGTGCTCGAGTCTA**TTACTTAAATAGCAACTCAGCAAATTGATT
*Dm*CE1B_nt	F	**CTTCCAGGGCCATAGT**ATCAGTCAAAATTATAAAAGTGTGCCG
	R	**TGGTGGTGCTCGAGTCTA**AATCCCGCTTGCCATACG
*Dm*CE1B_ct	F	**CTTCCAGGGCCATAGT**GAAGAAGCAGAGGTTGGCATTTC
	R	**TGGTGGTGCTCGAGTCTA**TTACTTAAATAGCAACTCAGCAAATTGATT
*Dm*CE1B_CBM48	F	**CTTCCAGGGCCATAGT**GAAGAAGCAGAGGTTGGCATTTC
	R	**TGGTGGTGCTCGAGTCTA**AATCCCGCTTGCCATACG
*Dm*CE6A	F	**CTTCCAGGGCCATAGT**CAGGATAAGTTTCATGTTTTTTATGTTTCG
	R	**TGGTGGTGCTCGAGTCTA**TTACTTCTTTAATGAATTTTCGTTATACAATTGCA

Table S6: Table of crystallographic statistics.

	*Dm*CE1B_ct	*Dm*CE1B_ct - MFA
Data Collection		
Date	March 27, 2020	June 8, 2020
Source	BioMAX at MAXIV	BioMAX at MAXIV
Wavelength (Å)	0.97625	0.97625
Space group	C121	$P12_11$
Cell dimensions		
a, b, c (Å)	103.96, 80.97, 107.35	73.26, 50.51, 90.15
α, β, γ (°)	90.0, 105.67, 90.0	90.0, 100.14, 90.0
No. of measured reflections	646182 (61524)	820122 (56295)
No. of independent reflections	94462 (9133)	123374 (10521)
Resolution (Å)	34.45 - 1.70 (1.76 - 1.70)	43.90 - 1.41 (1.46 - 1.41)
R_{merge} (%)	10.3 (143.7)	5.91 (59.8)
$CC_{1/2}$ (%)	99.8 (58.8)	99.9 (82.3)
Mean I/σI	9.34 (0.89)	16.5 (2.35)
Completeness (%)	99.6 (96.9)	98.2 (84.5)
Redundancy	6.8 (6.7)	6.6 (5.4)
Refinement		
R_{work}/R_{free}	0.168/ 0.211	0.161/0.179
No. atoms		
Protein	5622	5683
Ligand/ions	12	43
Water	648	723
B-factors		
Protein	34.0	19.7
Ligand/ions	41.3	29.5
Water	40.4	30.5
RMSD		
Bond length (Å)	0.007	0.009
Bond angles (°)	0.87	1.05
PDB accession	7B5V	7B6B

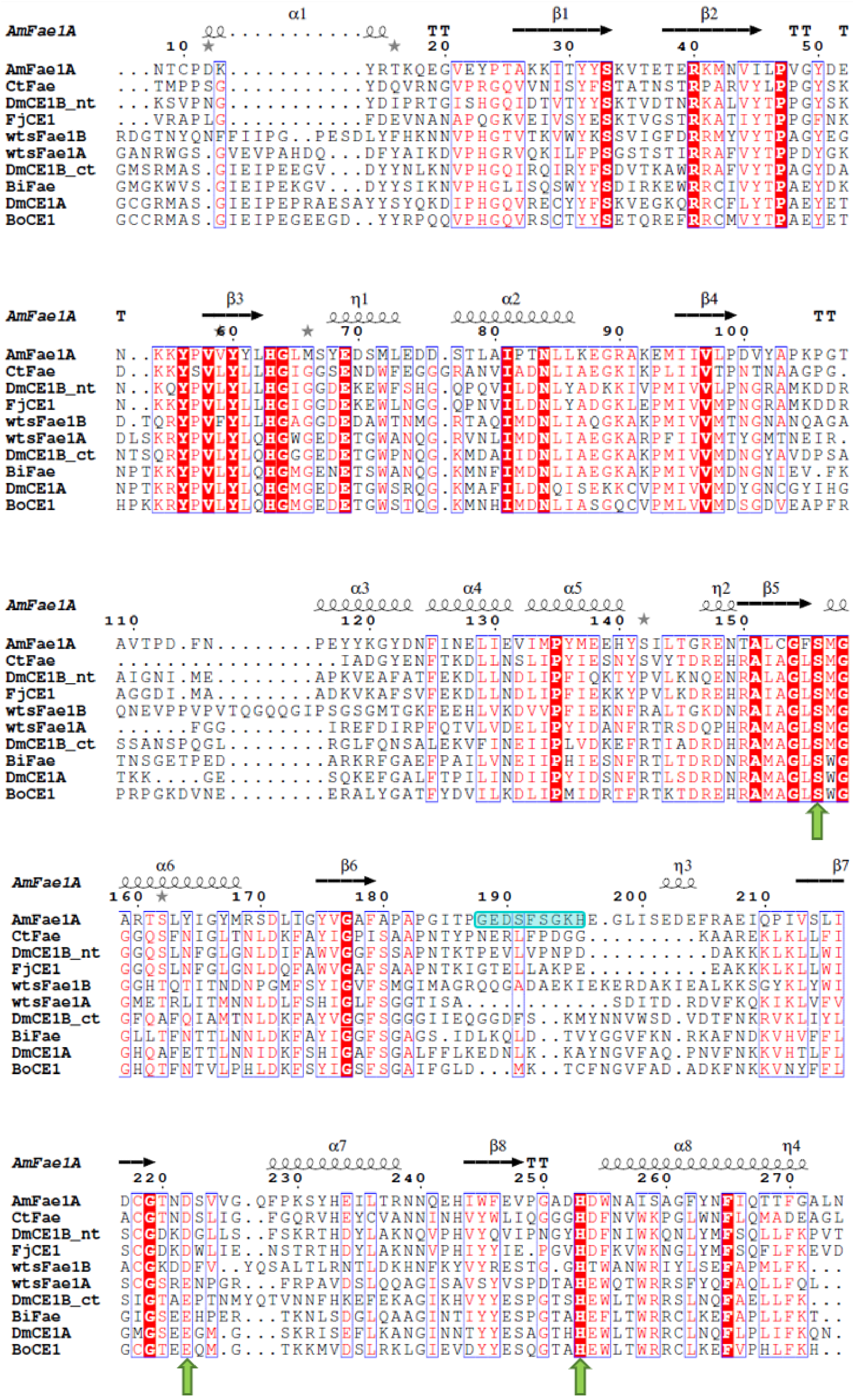
AmFae1A
α1 β1 β2 β3 η1 α2 β4 α3 α4 α5 η2 β5 α6 β6 η3 β7 α7 β8 α8 η4
AmFae1A
CtFae
DmCE1B_nt
FjCE1
wtsFae1B
wtsFae1A
DmCE1B_ct
BiFae
DmCE1A
BoCE1

Figure S1. Sequence alignment of the CE1 domains of *D. mossii* PUL 17 with related proteins. The secondary structural elements of *Am*Fae1A, a feruloyl esterase from *Anaeromyces mucronatus* (PDB accession: 5CXU; (3)) are shown above the alignment. The catalytic triad of *Am*Fae1A is indicated by green arrows and its *β*-clamp region is highlighted in cyan. Additional sequences included are from *Clostridium thermocellum* (*Ct*Fae; (4)), *F. johnsoniae* (*Fj*CE6; (5)), two esterases originating from a waste water sludge sample (wtsFae1A and wtsFae1B; (6)), *Bacteroides intestinalis* (*Bi*FAE; PDB accession 6MOT) and *B. ovatus* (*Bo*CE6; (5)). The alignment was created in Clustal Omega (7) and visualized using Espript 3.0 (8).

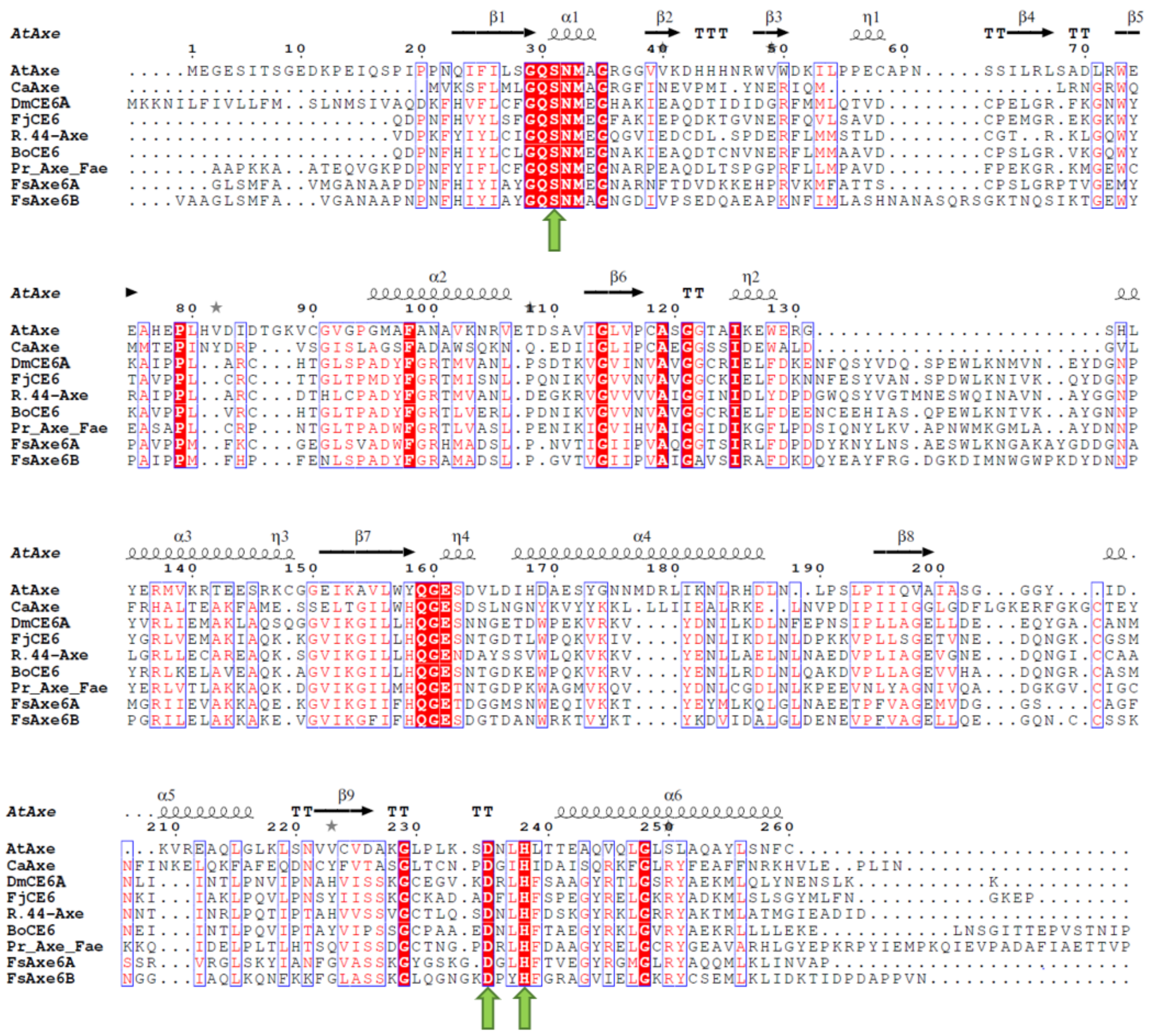

Figure S2. Sequence alignment of *Dm*CE6A with related proteins. The secondary structural elements of *At*Axe, a putative acetyl xylan esterase from *Arabidopsis thaliana* (PDB accession: 2APJ; (9)), are displayed above the alignment. The Ser-His-Asp catalytic triad of *At*Axe is indicated by green arrows. Additional sequences included are from *B. ovatus* (*Bo*CE6; (5)), *F. johnsoniae* (*Fj*CE6 and FjoAcCE; (5, 10)), *Prevotella ruminicola* (*Pr*Axe-Fae; (11)), *Fibrobacter succinogenes* (*Fs*Axe6A and *Fs*Axe6B; (12, 13)), a bovine rumen metagenome protein (R.44-Axe; (14, 15)), and *Clostridium acetobutylicum* (*Ca*Axe). The alignment was created in Clustal Omega (7) and visualized with Espript 3.0 (8).

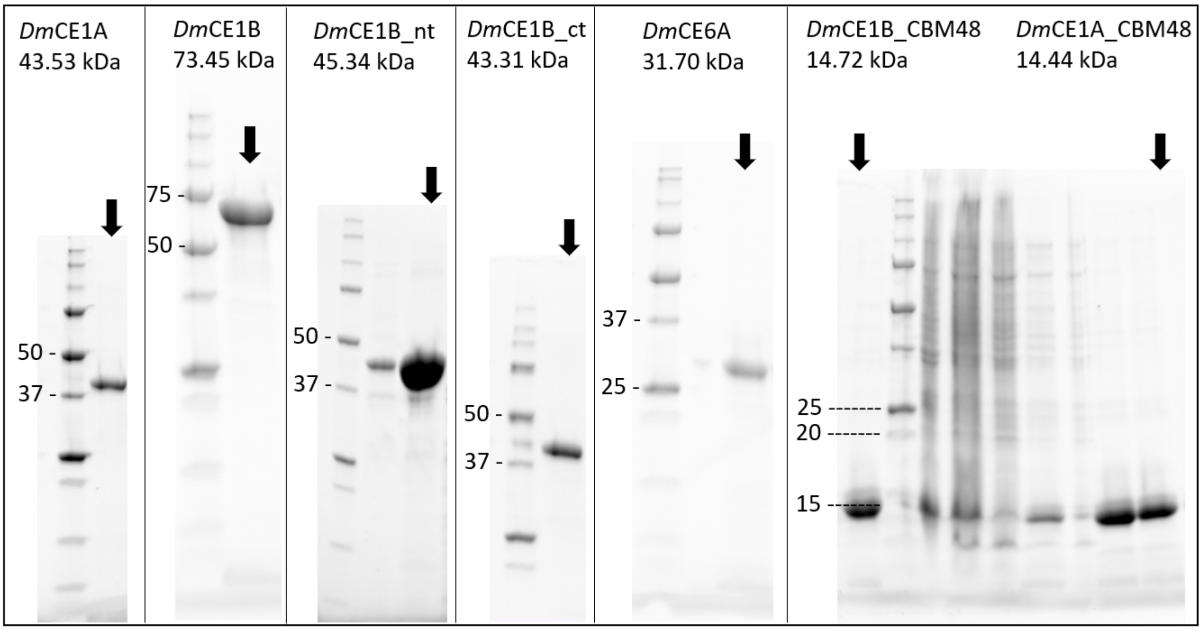

Figure S3. SDS-PAGE of produced proteins. The respective enzyme names and the expected molecular weights are given above each gel. The protein ladder (Precision Plus Protein^{TM} unstained Protein Ladder; Bio-Rad) is marked with relevant molecular weights. If several lanes on the gel are loaded with samples a black arrow marks the lane containing the relevant protein.

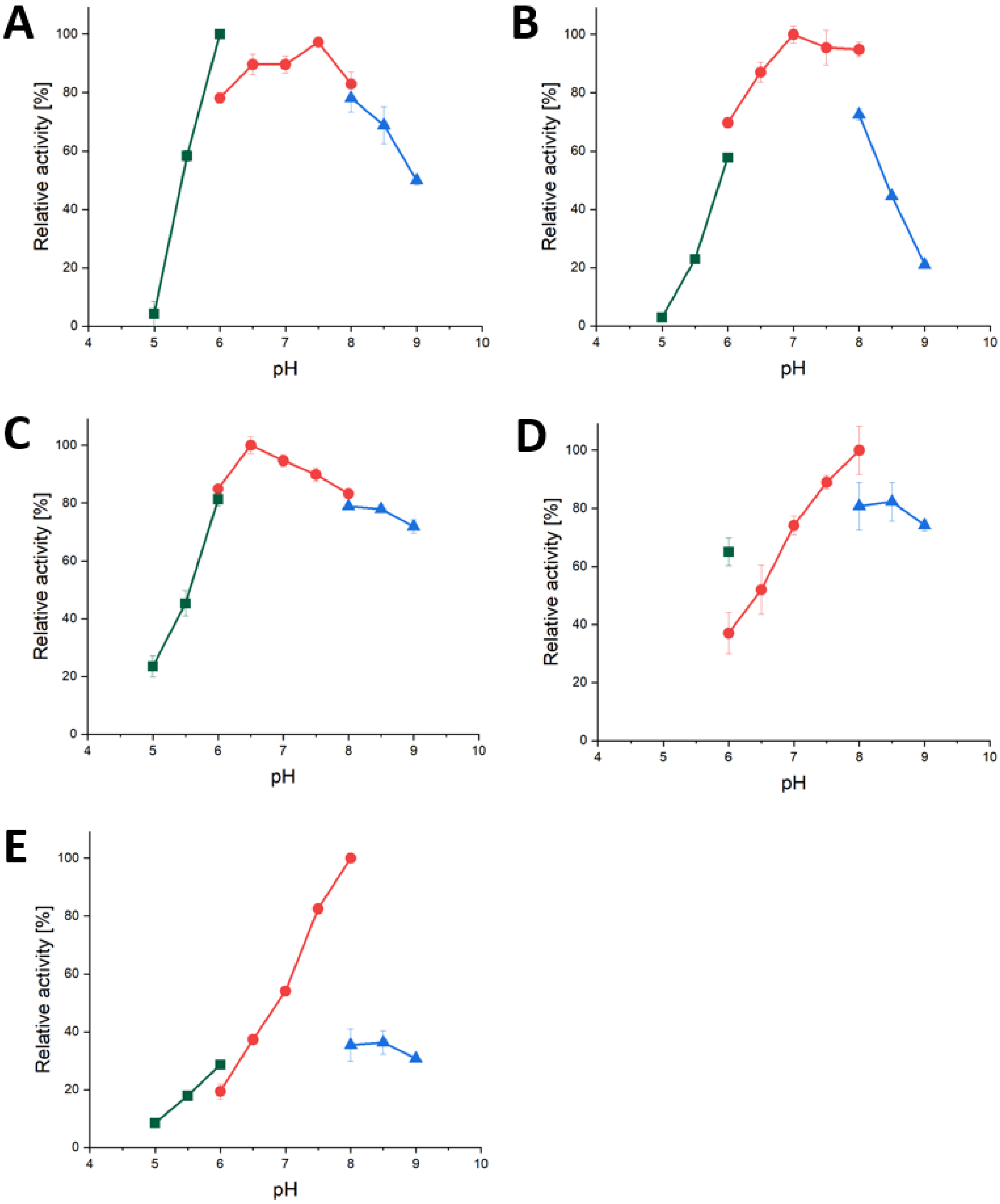

Figure S4. pH-dependent activity profiles of the *D. mossii* constructs. Here, 100% represents maximal activity for each individual enzyme. **(A)** *Dm*CE1A, **(B)** *Dm*CE1B, **(C)** *Dm*CE1B_nt, **(D)** *Dm*CE1B_ct and **(E)** *Dm*CE6A. pH profiles were determined by measuring activity on 4-MU-Ac (1 mM) using the buffers (100 mM): sodium citrate (green squares), sodium phosphate (red circles) and bicine (blue triangles). Results are averages of three experiments, and error bars represent one standard deviation.

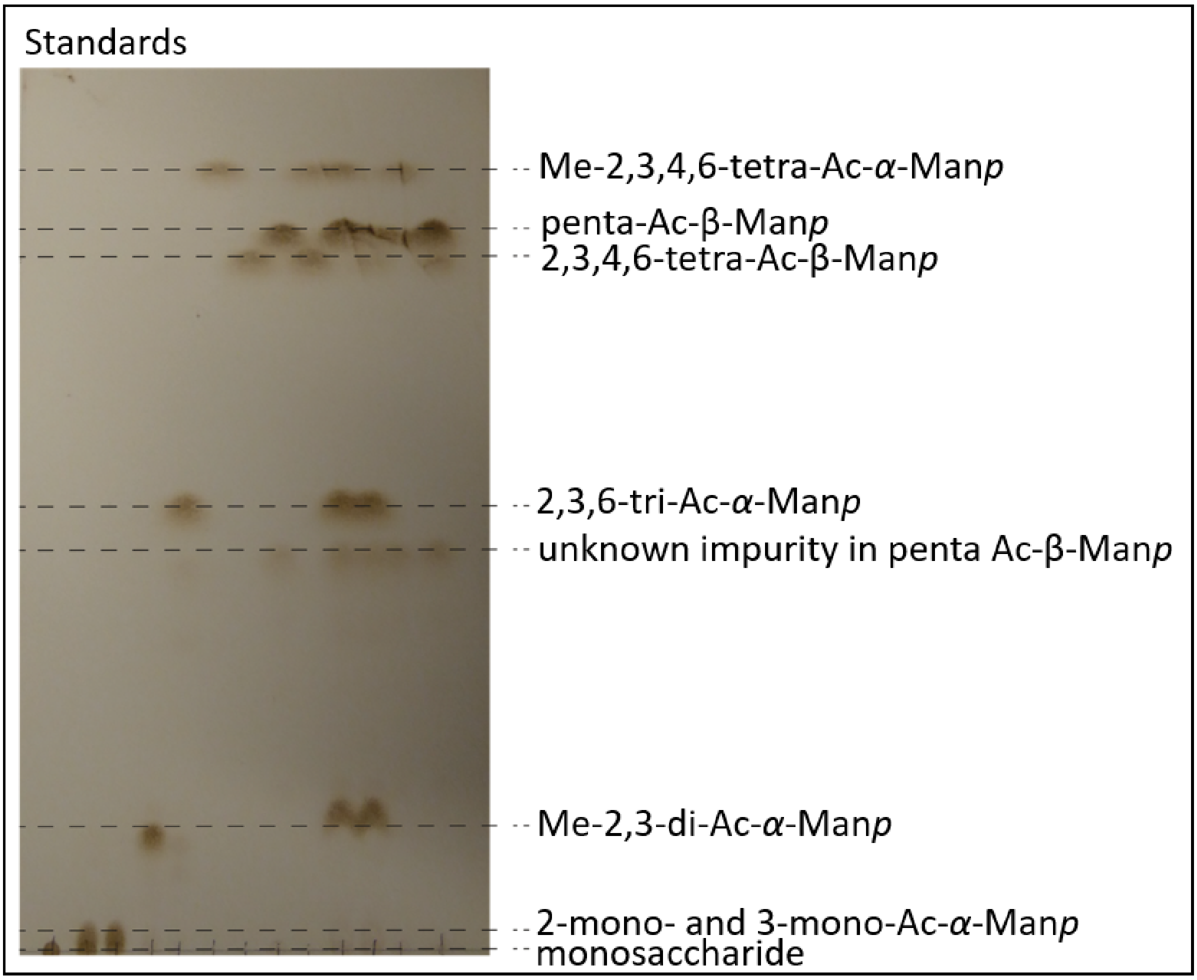

Figure S5. Validation of the TLC method showing effective separation and staining of a range of differently acetylated mannose monosaccharides. TLC was performed on aluminium-backed silica TLC plates using a 4:1 toluene:acetone mixture as running buffer. Standards were prepared at 20 mM.

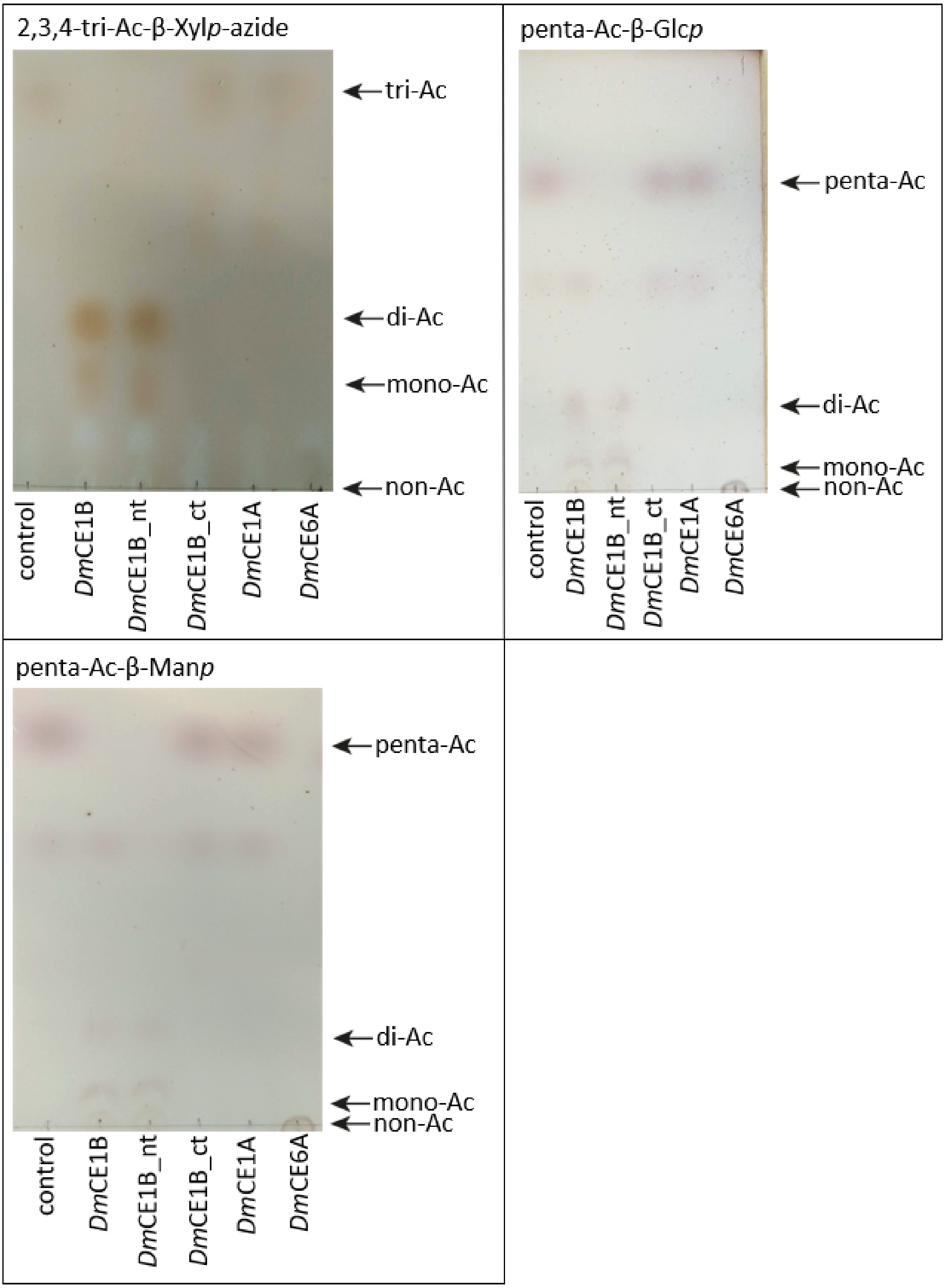

Figure S6. Activity of all enzyme variants on tri-acetylated *β*-xylose-azide, penta-acetylated *β*-glucose, and penta-acetylated *β*-mannose. Each substrate was spotted five times in the same position to allow for improved staining. Concentration of substrate was 5 mM.

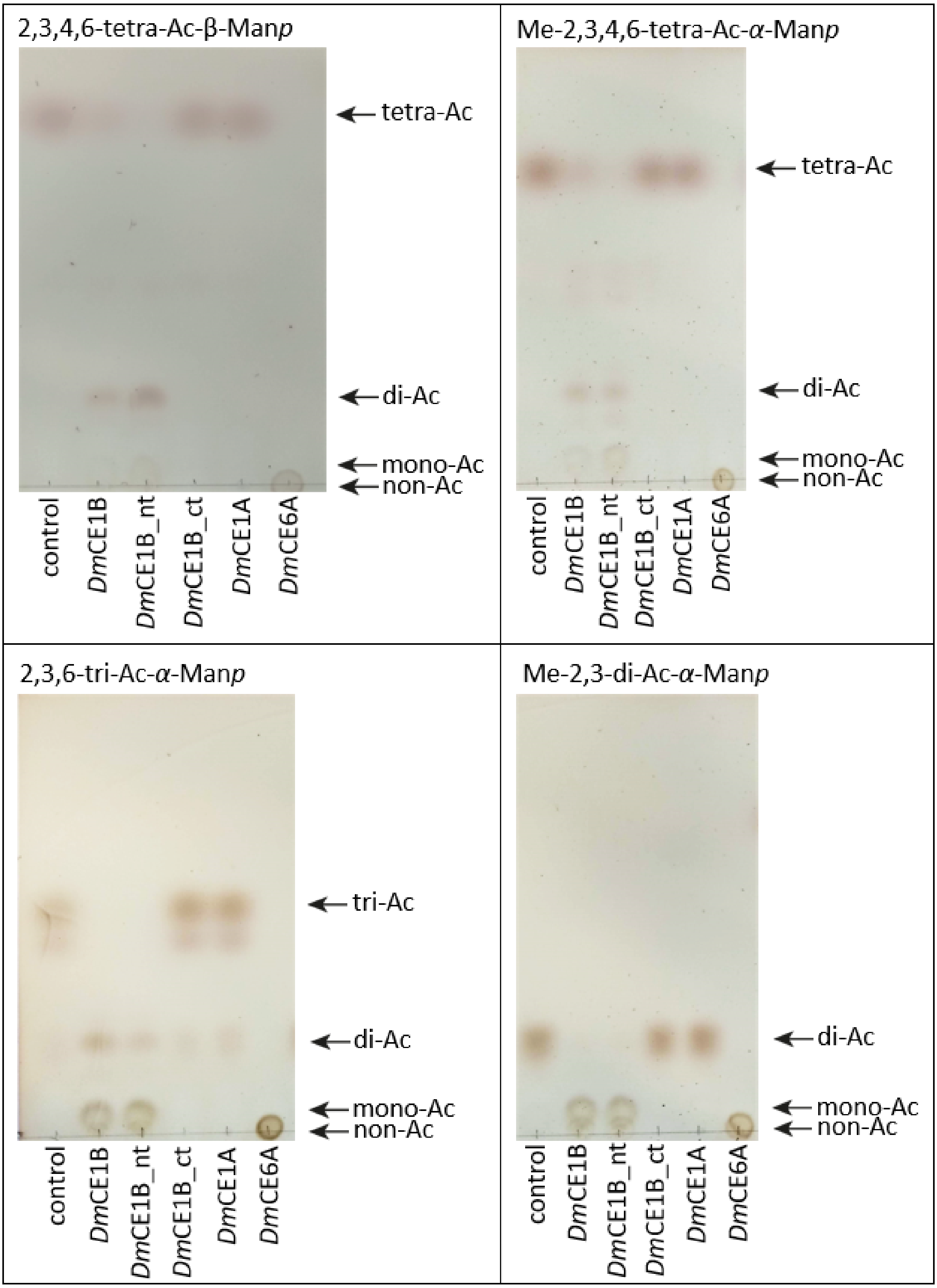

Figure S7. Activity of all enzyme variants on tetra-acetylated *β*-mannose, tetra-acetylated α-mannose, tri-acetylated *α*-mannose, and di-acetylated *α*-mannose. Each substrate was spotted five times in the same position to allow for improved staining. Concentration of substrate was 5 mM.

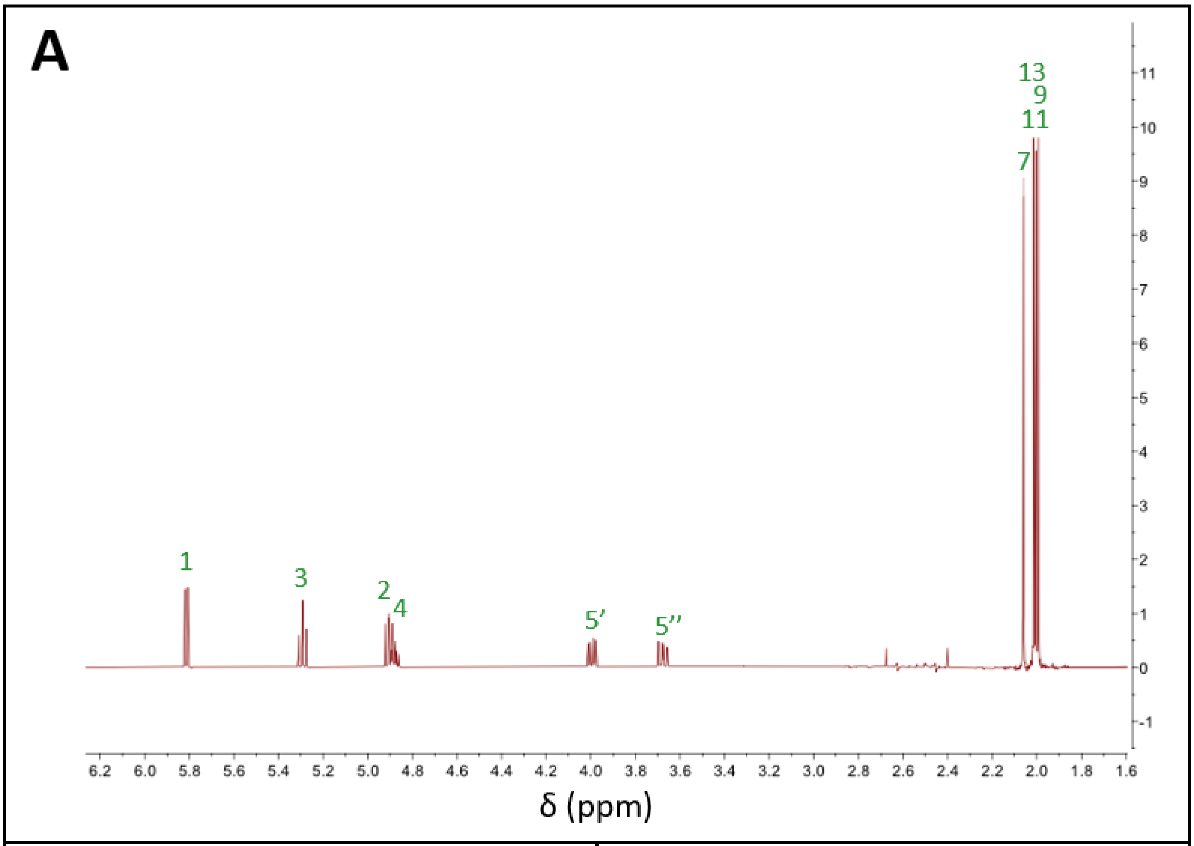

B

	δ_H	δ_C
1	5.81	91.43
2	4.90	69.33
3	5.29	70.5
4	4.89	68.06
5	3.68,3.99	61.92
6		168.82
7	2.06	21.43
8		169.08
9	2.01	21.23
10		169.41
11	1.99	21.30
12		169.52
13	2.00	21.42

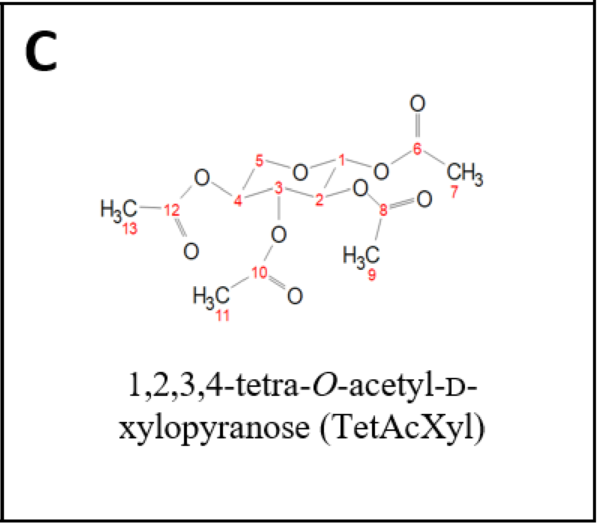

Figure S8. NMR studies of 1,2,3,4-tetra-*O*-acetyl-D-xylopyranose (TetAcXyl). (A) ^{1}H-NMR spectrum of TetAcXyl in DMSO-d6, with solvent suppressed**.** Assigned peaks are numbered. **(B)** Chemical shifts assigned to TetAcXyl. **(C)** Structure of TetAcXyl.

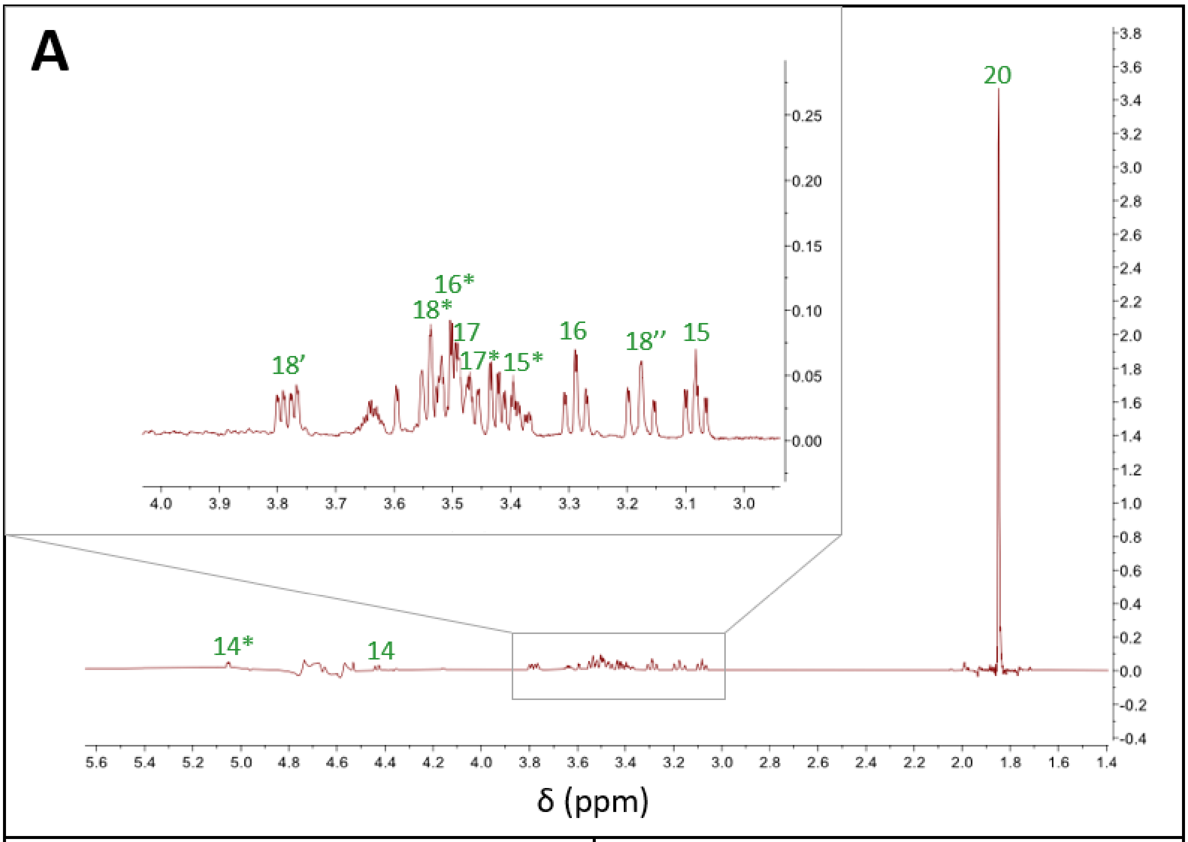

B

	δ_H	δ_C
14	4.42	96.64
14*	5.05	92.25
15	3.08	74.08
15*	3.39	71.44
16	3.29	75.84
16*	3.52	72.81
17	3.48	69.3
17*	3.47	69.23
18	3.17,3.77	65.1
18*	3.54	60.90
19		179.53
20	1.85	22.22

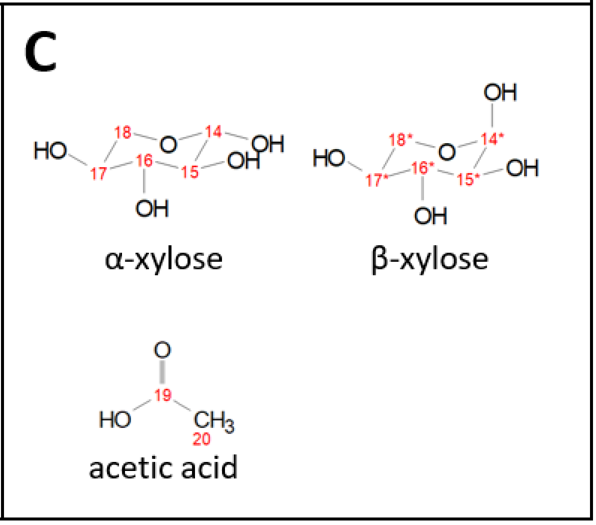

Figure S9. NMR studies of partly degraded TetAcXyl. (A) ^{1}H-NMR spectrum of TetAcXyl after incubation with *Dm*CE6A in D_2O, with solvent suppressed**.** Assigned peaks are numbered. **(B)** Chemical shifts assigned to α-xylose, β-xylose, and acetic acid **(C)** Structures of α-xylose, β-xylose, and acetic acid.

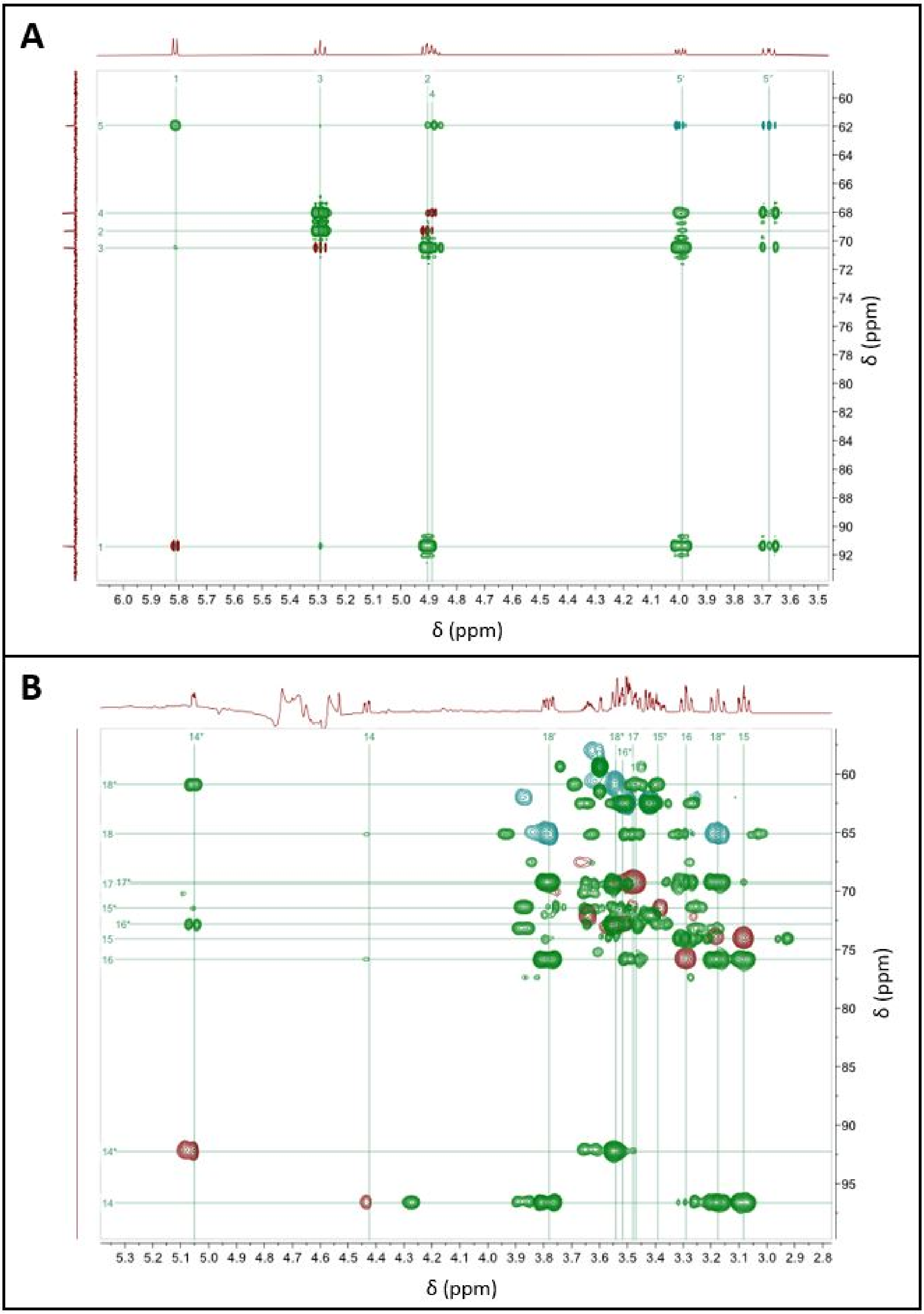
A
1
3
2
4
5'
5''
5
4
2
3
1
δ (ppm)
δ (ppm)
B
14'
14
18'
18''
17
16'
15''
16
18''
15
18''
18
17 17''
15''
16''
15
16
14''
14
δ (ppm)
δ (ppm)

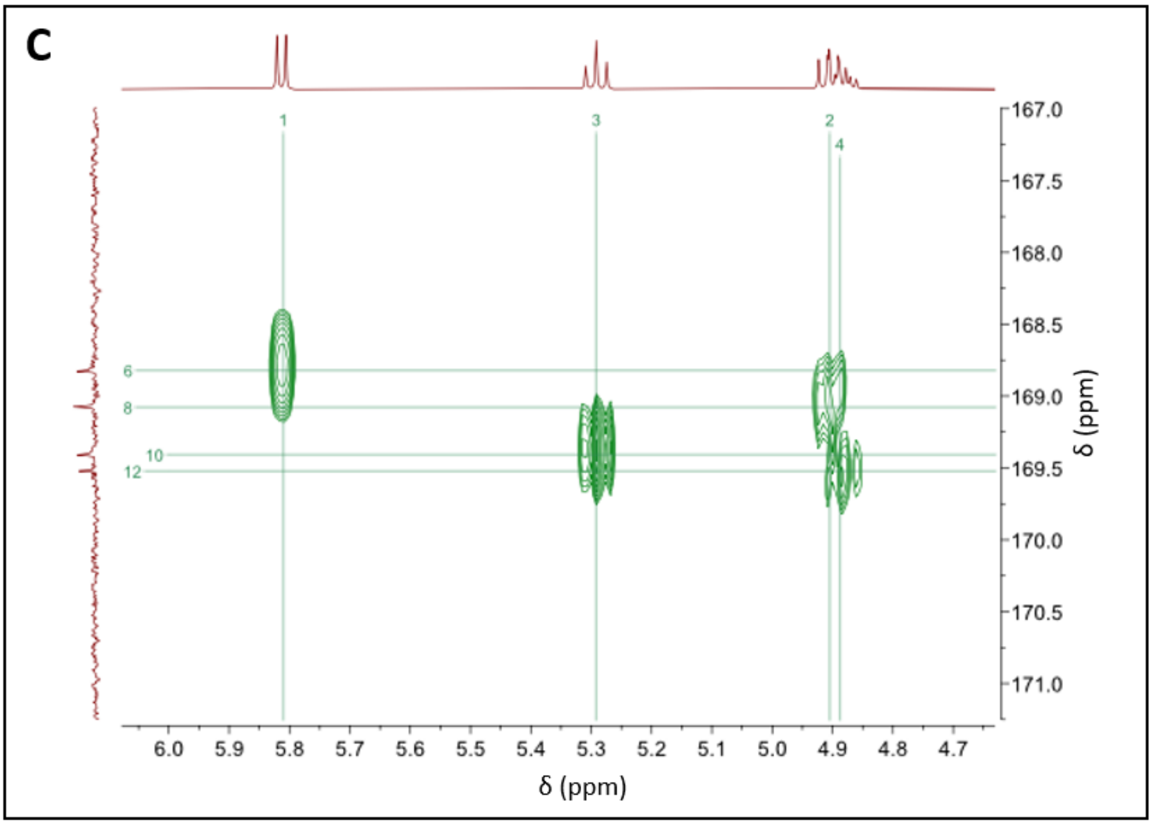

Figure S10. HSQC and HMBC spectra. Overlapped HSQC (red and turquoise) and HMBC (green) spectra of **(A)** 1,2,3,4-tetra-*O*-acetyl-D-xylopyranose (TetAcXyl) and **(B)** TetAcXyl partly degraded by *Dm*CE6A, showing mainly signals originating from xylose, but also signals originating from a shorter lived intermediate. **(C)** HMBC spectrum of TetAcXyl showing the correlations between xylose protons and carbons on the acetate group.

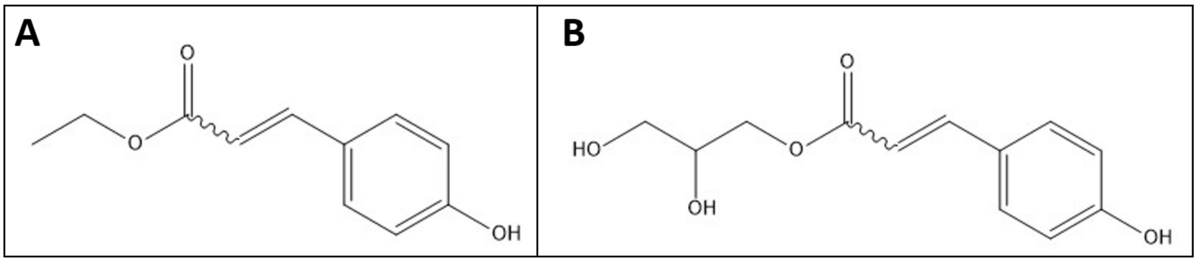

Fig. S11: The structures of *p*-coumaric ethyl ester **(A)** and *p*-coumaric 2,3-dihydropropyl ester **(B)**.

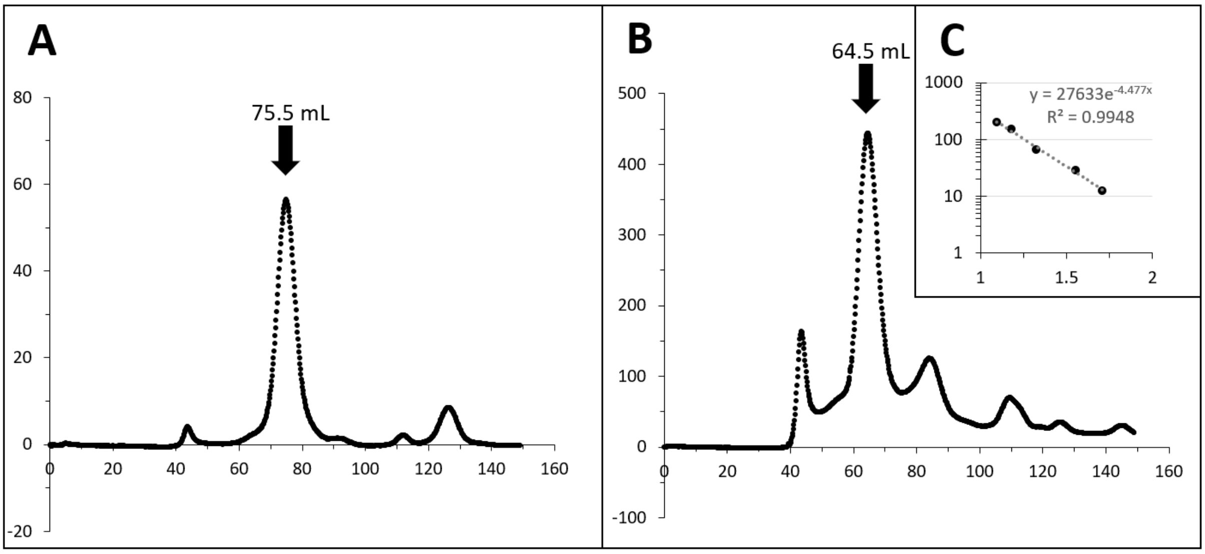

Fig. S12. Size exclusion chromatograms of **(A)** *Dm*CE1B_ct and **(B)** *Dm*CE1B. **(C)** shows the standard curve including cytochrome c from horse heart (12.4 kDa), carbonic anhydrase from bovine erythrocytes (29 kDa), bovine serum albumin (66 kDa), alcohol dehydrogenase from yeast (150 kDa), *β*-amylase from sweet potato (200 kDa) and blue dextran (2000 kDa). Protein elution peaks are labelled with black arrows and their respective elution volume. The calculated size of *Dm*CE1B_ct is 66.8 kDa. As the calculated molecular mass is notably bigger than the expected size (43.31 kDa) dimerization is likely. The calculated size of *Dm*CE1B is 159.4 kDa which roughly corresponds to double of the molecular weight of *Dm*CE1B (73.45 kDa), again indicating dimerization.

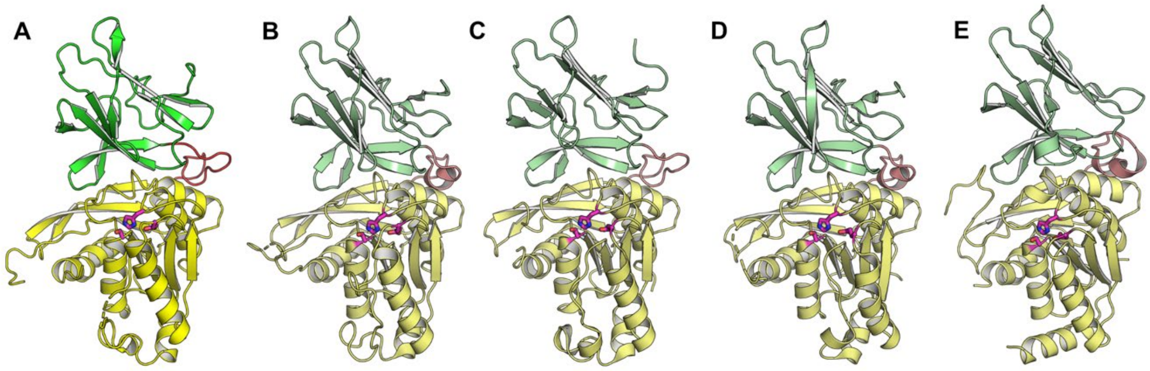

Fig. S13. Comparison of CBM48-CE1 structures. **(A)** *Dm*CE1B_ct, **(B)** putative Fae (Bacint_01039) from *B. intestinalis* (PDB: 6NE9), **(C)** putative Fae (Bacint_01033) from *B. intestinalis* (PDB: 6MOU), **(D)** wtsFae1A (PDB: 6RZO), and **(E)** wtsFae1B (PDB: 6RZN). *Dm*CE1B_ct is colored as in Fig. 3 where the CBM48 domain is colored green, the linker is colored red, and the CE1 domain is colored yellow. The equivalent domains in the other proteins are shown in pale colors, and the residues of the catalytic triad are colored in magenta in each.

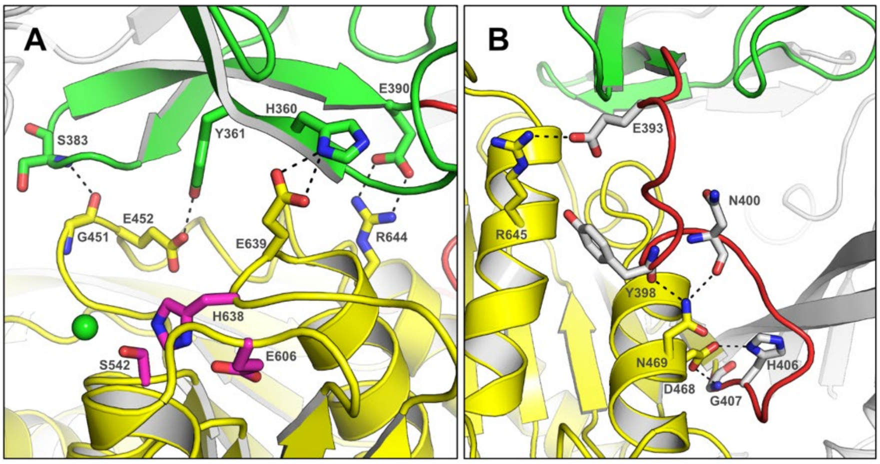

Figure S14. Interactions at the domain interfaces of *Dm*CE1B_ct. Interactions made between (A) the CE1 and the CBM48 domains, and between (B) the CE1 domain and the linker region are shown. The CE1 domain, CBM48 domain, and linker region are coloured yellow, green, and red, respectively. Residues making interactions at the interface of the domains are shown as sticks, with the residues of the linker region colored in grey for clarity. The catalytic triad residues are shown as sticks and colored magenta and the chloride ion observed in the active site pocket is shown as a green sphere.

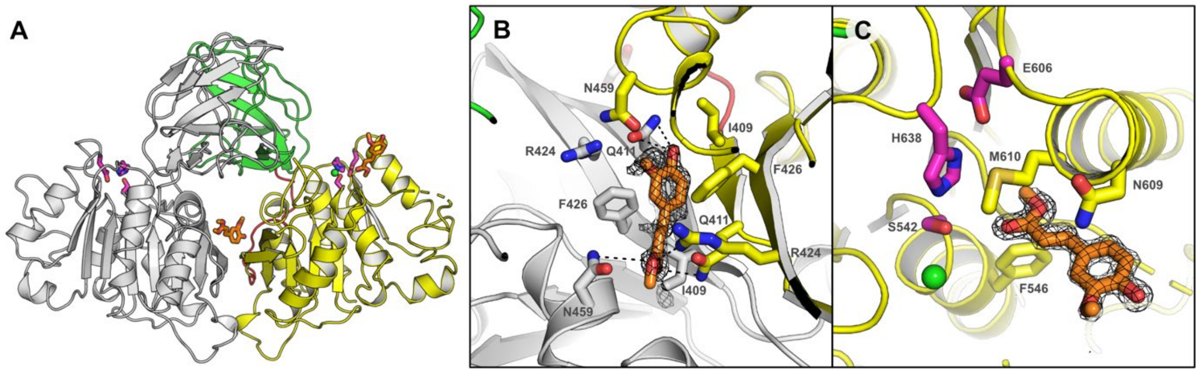

Figure S15. *Dm*CE1B_ct in complex with MFA. **(A)** Overall structure of *Dm*CE1B_ct in complex with MFA showing the MFA ligand in orange sticks bound at two sites on the protein. **(B)** Binding of MFA at the site between the two protein molecules and the residues lining the binding site. **(C)** Binding of MFA near the active site. Electron density for the MFA molecules, generated by an omit map and carved at 2Å and displayed at 3σ, are shown as mesh.

References

1. Dührkop, K., Fleischauer, M., Ludwig, M., Aksenov, A. A., Melnik, A. V., Meusel, M., Dorrestein, P. C., Rousu, J., and Böcker, S. (2019) SIRIUS 4: A rapid tool for turning tandem mass spectra into metabolite structure information. *Nat. Methods*. **16**, 299–302
2. Böcker, S., and Dührkop, K. (2016) Fragmentation trees reloaded. *J. Cheminform.* **8**, 1–26
3. Gruninger, R. J., Cote, C., Mcallister, T. A., and Abbott, D. W. (2016) Contributions of a unique β-clamp to substrate recognition illuminates the molecular basis of exolysis in ferulic acid esterases. *Biochem. J.* **437**, 839–849
4. Schubot, F. D., Kataeva, I. A., Blum, D. L., Shah, A. K., Ljungdahl, L. G., Rose, J. P., and Wang, B. C. (2001) Structural basis for the substrate specificity of the feruloyl esterase domain of the cellulosomal xylanase Z from *Clostridium thermocellum*. *Biochemistry*. **40**, 12524–12532
5. Kmezik, C., Bonzom, C., Mazurkewich, S., Olsson, L., and Larsbrink, J. (2020) Multimodular fused acetyl-feruloyl esterases from soil and gut Bacteroidetes improve xylanase depolymerization of recalcitrant biomass. *Biotechnol. Biofuels*. **13**, 1–14
6. Holck, J., Fredslund, F., Møller, M. S., Brask, J., Krogh, K. B. R. M., Lange, L., Welner, D. H., Svensson, B., Meyer, A. S., and Wilkens, C. (2019) A carbohydrate-binding family 48 module enables feruloyl esterase action on polymeric arabinoxylan. *J. Biol. Chem.* **294**, 17339–17353
7. Madeira, F., Park, Y. mi, Lee, J., Buso, N., Gur, T., Madhusoodanan, N., Basutkar, P., Tivey, A. R. N., Potter, S. C., Finn, R. D., and Lopez, R. (2019) The EMBL-EBI search and sequence analysis tools APIs in 2019. *Nucleic Acids Res.* **47**, 636–641
8. Robert, X., and Gouet, P. (2014) Deciphering key features in protein structures with the new ENDscript server. *Nucleic Acids Res.* **42**, 320–324
9. Bitto, E., Bingman, C. A., McCoy, J. G., Allard, S. T. M., Wesenberg, G. E., and Phillips Jr, G. N. (2005) The structure at 1.6 Angstroms resolution of the protein product of the At4g34215 gene from Arabidopsis thaliana. *Acta Crystallogr. Sect. D*. **61**, 1655–1661
10. Razeq, F. M., Jurak, E., Stogios, P. J., Yan, R., Tenkanen, M., Kabel, M. A., Wang, W., and Master, E. R. (2018) A novel acetyl xylan esterase enabling complete deacetylation of substituted xylans. *Biotechnol. Biofuels*. 10.1186/s13068-018-1074-3
11. Kabel, M. A., Yeoman, C. J., Han, Y., Dodd, D., Abbas, C. A., Bont, J. A. M. De, Morrison, M., Cann, I. K. O., and Mackie, R. I. (2011) Biochemical characterization and relative expression levels of multiple carbohydrate esterases of the xylanolytic rumen bacterium *Prevotella ruminicola* 23 grown on an ester-enriched substrate. *Appl. Environ. Microbiol.* **77**, 5671–5681
12. Kam, D. K., Jun, H.-S., Ha, J. K., Inglis, D. G., and Forsberg, C. W. (2005) Characteristics of adjacent family 6 acetylxylan esterases from *Fibrobacter succinogenes* and the interaction with Xyn10E xylanase in the hydrolysis of acetylated xylan. *Can. J. Microbiol.* **51**, 821–832
13. Yoshida, S., Mackie, R. I., and Cann, I. K. O. (2010) Biochemical and domain analyses of FSUAxe6B, a modular acetyl xylan esterase, identify a unique carbohydrate binding module in *Fibrobacter succinogenes* S85. *J. Bacteriol.* **192**, 483–493
14. López-Corés, N., Reyes-Duarte, D., Beloqui, A., Polaina, J., Ghazi, I., Golyshina, O. V, Ballesteros, A., Golyshin, P. N., and Ferrer, M. (2007) Catalytic role of conserved HQGE motif in the CE6 carbohydrate esterase family. *FEBS Lett.* **581**, 4657–4662
15. Ferrer, M., Golyshina, O. V, Chernikova, T. N., Khachane, A. N., Reyes-Duarte, D., Martins Dos Santos, V. A. P., Strompl, C., Elborough, K., Jarvis, G., Neef, A., Yakimov, M. M., Timmis, K. N., and Golyshin, P. N. (2005) Novel hydrolase diversity retrieved from a metagenome library of bovine rumen microflora. *Environ.* **7**, 1996–2010
